# A screen for constituents of motor control and decision making in *Drosophila* reveals visual distance-estimation neurons

**DOI:** 10.1038/srep27000

**Published:** 2016-06-03

**Authors:** Tilman Triphan, Aljoscha Nern, Sonia F. Roberts, Wyatt Korff, Daniel Q. Naiman, Roland Strauss

**Affiliations:** 1 Janelia Research Campus, Howard Hughes Medical Institute, 19700 Helix Drive, Ashburn, VA 20147, USA; 2 Johns Hopkins University, Department of Applied Mathematics and Statistics, 3400 North Charles Street, Baltimore, MD 21218, USA; 3 Johannes Gutenberg-Universität Mainz, Institut für Zoologie III, Col.-Kleinmann-Weg 2, 55099 Mainz, Germany

## Abstract

Climbing over chasms larger than step size is vital to fruit flies, since foraging and mating are achieved while walking. Flies avoid futile climbing attempts by processing parallax-motion vision to estimate gap width. To identify neuronal substrates of climbing control, we screened a large collection of fly lines with temporarily inactivated neuronal populations in a novel high-throughput assay described here. The observed climbing phenotypes were classified; lines in each group are reported. Selected lines were further analysed by high-resolution video cinematography. One striking class of flies attempts to climb chasms of unsurmountable width; expression analysis guided us to C2 optic-lobe interneurons. Inactivation of C2 or the closely related C3 neurons with highly specific intersectional driver lines consistently reproduced hyperactive climbing whereas strong or weak artificial depolarization of C2/C3 neurons strongly or mildly decreased climbing frequency. Contrast-manipulation experiments support our conclusion that C2/C3 neurons are part of the distance-evaluation system.

Terrestrial locomotion is an important mode of translocation for *Drosophila melanogaster* flies as searching for food in the near-field and courtship occur during walking. A striking aspect of walking behaviour is the crossing of gaps of up to 1.7-times the fly’s body length of 2.6 mm. Unmistakable leg-over-head strokes of the forelegs indicate an attempt. Therefore, turning back without trying to climb can reliably be distinguished from a futile attempt^1^. The probability for a climbing attempt is gap-width dependent. Gaps smaller than body size are usually overcome just by a large stride. Climbing behaviour becomes frequent at broader, yet surmountable gaps; then the initiation rate decreases with increasing width. Flies estimate gap width during approach by the parallax motion they perceive from the other side of the gap, not by binocular depth perception^1^. Distance estimation to objects in *Drosophila* relies on small-field motion vision^2^; parallax motion but not looming of retinal images is used while walking. These picture shifts between foreground and background contain distance information because the distal side’s image of a narrow gap moves faster over the retina than that of a wide gap. Gap-crossing behaviour is not just remarkable for this visually guided decision process, but also for its precise execution, requiring integration of mechanosensory, proprioceptive and visual information^1^,^3^. Forelegs stretch out far to reach the distal side of the gap, middle legs lift up the body to a vertical position within the gap, and hind legs are positioned as close as possible to the gap’s proximal edge. No legged robot is as dexterous as these climbing flies. Understanding the neuronal control of gap crossing is therefore of applied interest in addition to providing insights into general biological processes such as decision making. However, the neuronal circuitry underlying climbing behaviour is largely unknown.

Here, we report design and results of a neuronal inactivation screen aimed at discovering brain regions and circuit components controlling gap-crossing behaviour. Previous studies were performed on single flies under high-speed cameras^1^,^3^, a time-consuming procedure. To test for decision making and climbing execution in a high-throughput way, we devised a ring-gap assay with automated evaluation, in which the flies are faced with concentric, water-filled gaps of increasing width. Wild-type flies emerging from a centre recess explore the climbing disk even in the absence of attractive stimuli; they surmount wider and wider gaps until most of them decide not to climb over the outermost 4.0-mm-wide ring-gap. The cardinal heading is radially outward, because randomly searching flies increase the straight path increments between consecutive turns over time so that they are exploring larger and larger areas^4^,^5^. Fifteen flies per run were video-recorded for 10 min, permitting quantitative analyses of their decision-making and climbing abilities.

We screened 2,415 R-GAL4 lines; all genetic^6^ and expression-pattern data are available^7^. To conditionally interrupt synaptic transmission of neurons labelled by those drivers, lines were crossed to UAS-*shibire*^*ts*^ (*shi*^*ts*^)^8^, encoding a temperature-sensitive, dominant-negative form of dynamin. Flies were raised with functional dynamin (21.6 °C) and tested in the ring-gap assay with disrupted endocytosis (34 °C).

The screen revealed lines with various forms of altered climbing which were categorized. Selected lines were analysed by high-speed videography, which supported the primary-screen classification and revealed clues to the nature of individual defects. Expression patterns of GAL4 lines revealed candidate cell types for many phenotypes, but the complexity of most patterns precluded direct conclusions about the neurons responsible. Follow-up experiments are therefore required to determine the neuronal basis of phenotypes of interest. Here we scrutinized a line with increased number of climbing attempts at large and even insurmountable gaps. This phenotype could reflect deficits in decision making or visual distance estimation informing climbing decisions. The line showed expression in a specific population of optic-lobe feedback neurons. Inactivation of just these C2 cells and/or the closely related cell-type C3 with existing^9^ and newly generated split-GAL4 lines reproduced the behavioural phenotype. Conversely, artificial depolarization of C2 and/or C3 neurons by expressing the temperature-sensitive ion channel^10^ *dTrpA1* decreased climbing activity. Finally, under conditions where perceivable parallax motion was strongly reduced, activation or silencing of C2 neurons had little effect on climbing behaviour. If perceivable parallax motion was optically enhanced, activation or silencing of C2 neurons had strong and antagonistic effects. These findings show that altered visual perception via C2/C3 manipulation is sufficient to shift a fly’s climbing decisions to no longer match the actual gap width and demonstrate a role of C2/C3 cells in distance-estimation processing from parallax motion.

## Results

### Primary Screen

**Figure 1:**
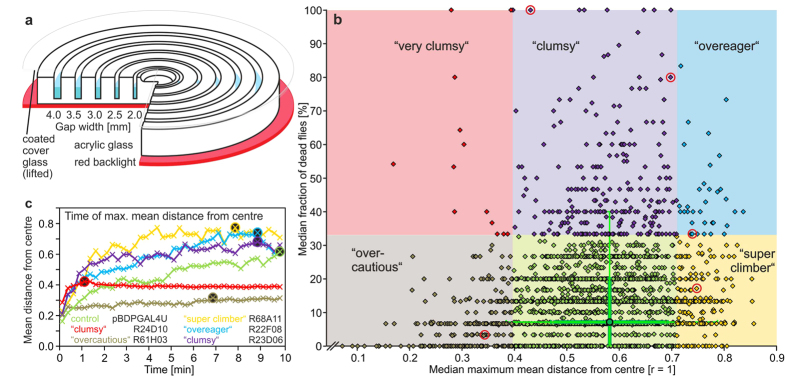
Diagram of the ring-gap climbing assay and performance of 2,415 GAL4 lines driving UAS-*shibire*^*ts*^ to inactivate sets of neurons at 34 °C. (**a**) Mattfinished Perspex climbing disk with five concentric ring-gaps of 2.0 to 4.0 mm widths (in steps of 0.5 mm) and a bowl-like centre recess milled into it. Gaps are filled half-high with water plus detergent. Fifteen anesthetized flies of a given line are placed into the recess and a coated glass plate is lowered onto the disk surface. The cover glass is lifted by 3.0 mm when all flies are awake. Flies can distribute for 10 min; an overhead camera records their whereabouts once a 1s; the red backlight improves visibility. (**b**) Each diamond of the scatter plot represents the median outcome of 3 to 32 replications per line in sets of 15 flies (16,189 experiments; Supplementary Table 1. The median maximum of the mean distance from centre of the 15 flies reached any time during the 10-min recording is represented on the x-axis (in relative units of the radius of the disk [0, centre to 1, outer rim]) whereas the y-axis reflects the median percentage of dead flies after 10 min. In terms of the median maximum mean distance from centre the lowest 10% of all R-lines are classified as under-performers and the uppermost 10% as over-performers. We further classified the R-lines into groups with either normal or significantly increased rate of dead flies. The boundary was chosen at 10% of the R-lines with the most losses. These definitions divide the R-lines into 6 classes. The centre of the green cross represents, in both dimensions, the medians of 1,412 control experiments, each comprising 15 flies of pBDPGAL4U driving UAS-*shi*^*ts*^ at 34 °C. Example lines of Figs 1c and 2 are marked by red rings. The thick lines represent the 25%-/75%-quartiles, and the thin lines the 10%-/90%-quantiles. (**c**) Examples of the development of the mean distance from centre of 15 flies per experiment over time (relative units; 0, centre and 1, rim of the disk). Maximum mean distances are indicated by circles.

In the ring-gap assay (Fig. 1a) 15 male flies of a given genotype get 10 min to explore a disk with five concentric, water-filled gaps of increasing width (2.0, 2.5, 3.0, 3.5, 4.0 mm). Flies start from a centre recess after a glass lid has been lifted from the disk surface by 3 mm. This low ceiling prevents flight and jumping so that wings can stay intact. When flies failed in surmounting a gap and fell, they quickly drowned and thereby marked their smallest challenging gap width. The outermost gap particularly demands the width-estimation capabilities of the flies. Controls usually refrain from attempting to climb this challenging gap size (Video 1). Each genotype was tested at least three times. We interspersed experiments on R-GAL4 lines crossed to UAS-*shi*^*ts*^ with controls, an R-line with P-insert but no expression. pBDPGAL4U > UAS-*shi*^*ts*^ flies at the temperature of the experiment have previously been used as controls in R-line screens^11^,^12^,^13^. Testing locomotor behaviour of R-GAL4 > UAS-*shi*^*ts*^–flies at the restrictive and the permissive temperature would superimpose temperature-owed differences.

We performed 16,189 experiments on 2,415 different R-lines and 1,412 experiments on pBDPGAL4U > UAS-*shi*^*ts*^ controls. The numbers of dead flies in the five grooves and all kinematic data (see Supplementary videos) were evaluated off-line. The distance of each fly from the centre of the disk and the mean distance for the 15 flies were calculated once per second (Fig. 1c). Dead flies kept contributing to the mean distance with their ultimate position in one of the grooves. The maximum of these mean distances at any time during the 10-min-test period served as one main classification parameter. The median of the maximum mean distances of all repetitions for a given line are shown on the x-axis in Fig. 1b. The other parameter is the final number of dead flies in the grooves, plotted as median per line on the y-axis. The distribution of all pBDPGAL4U > UAS-*shi*^*ts*^ control experiments is summarized in the green cross, the centre of which represents the medians of both parameters. The coloured fields indicate the lowest and highest 10% of R-lines regarding maximum mean distance from centre and the 10% with the highest percentage of dead flies. There was no statistically meaningful lower rate of dead flies in comparison to controls. Using these guidelines, the majority of R-lines can be found in the normal range (69.8%; green area; normal climbing behaviour with low death rate) in good agreement with the performance of the control flies. Flies of some of the R-lines in the brown area of Fig. 1b (9.5%; “overcautious”) do not even leave the centre recess; all others manage to overcome just the smaller gaps. The death rate in this group is within the normal range; most flies survive until the end of the experiment. The red area (0.5%; “very clumsy”) comprises R-lines that drown already in the small grooves. The purple area (10.1%; “clumsy”) is populated by R-lines with high death rates but normal climbing success. The blue area (1.4%; “overeager”) represents R-lines, which get out further into the periphery of the disk than the majority of all lines, but at the cost of a high death rate. Most of these losses occur at the broad gaps (3.5 and 4.0 mm). Finally, the yellow area (8.6%; “super climber”) represents R-lines with flies surmounting even the broadest 4.0 mm gaps more frequently than the majority of lines with no increase in death rate. The group names serve as quick reference tool, psychological connotations are not intended. At this first stage of analysis, an “overcautious” line may simply be incapacitated. The line names are listed by hit category in Supplementary Table 1. A comparative view into the time development of the mean distances of the 15 flies from centre reveals kinematic differences in the categories (Fig. 1c). The “super-climber” example flies R68A11 > *shi*^*ts*^ (yellow) move out quickest. This is true also for flies of the “overeager” example R22F08 > *shi*^*ts*^ (blue), just that those flies fail at the wider gaps. R24D10 > *shi*^*ts*^ (red) flies as an example of a “clumsy” line close to the “very clumsy” boundary move out quickly but fail already at the small-size gaps (Video 2). In contrast, all “overcautious” flies from R61H03 > *shi*^*ts*^ (brown) stay alive but basically because they do not move much (Video 3). Further kinematic parameters calculated from screen data are given in Supplementary Fig. S1.

### In-Depth Analysis by High-Speed Cinematography

**Figure 2:**
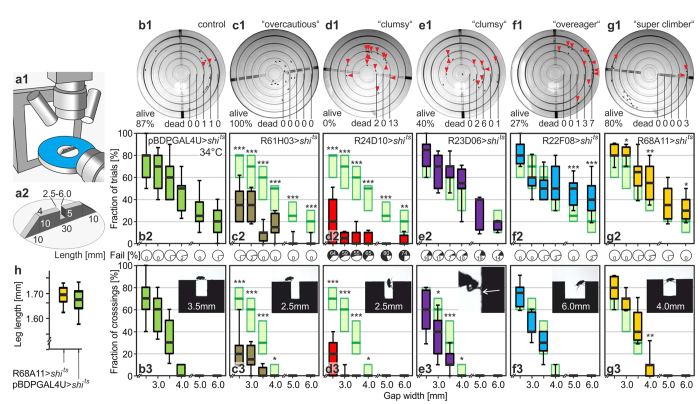
High-speed video analysis data of the example lines shown in Fig. 1. (**a1)** Shows a sketch of the setup and (**a2**) centre details. A catwalk with ramps on both sides and the gap in the middle is situated on a water-surrounded platform. Side and top views of climbing flies are taken at 200 fps against a LED-backlight and under LED-downlight, respectively. (**b1–g1**) Overhead camera shots at t = 10 min, the end of the experiments (the drilled pipe for filling the gaps can be seen). Arrowheads and numbers indicate dead flies in the grooves. (**b2–g2**) Fraction of climbing trials with percentage of falling, and (**b3**–**g3**) fraction of successful crossings, respectively, both per 10 approaches to the gap per fly at 34 °C. N  ≥ 10 flies per line and per two widths. Lines were tested on surmountable (2.5 to 4.0 mm) and insurmountable widths (5.0 and 6.0 mm) to test climbing and width-estimation capabilities. Boxes indicate 25%-/75%-quartiles, thick lines medians. Whiskers indicate 10%-/90%-quantiles. Insets show typical side views; successful crossing of a demanding gap width (**b3**) by a pBDPGAL4U > *shi*^*ts*^ control fly, (**c3**) failure to initiate crossing at an easy gap width by a R61H03 > *shi*^*ts*^ fly, (**d3**) failure in surmounting an easy gap by a R24D10 > *shi*^*ts*^ fly, (**e3**) malfunction of the tarsi (arrow) by a R23D06 > *shi*^*ts*^ fly, (**f3**) fruitless attempt by a R22F08 > *shi*^*ts*^ fly at a gap of insurmountable width, and (**g3**) successful crossing of a gap of just surmountable width by a R68A11 > *shi*^*ts*^ fly. The latter flies perform better than controls; median leg lengths (**h**) n = 14; femur + tibia + tarsi) proof that they are not bigger than pBDPGAL4U > *shi*^*ts*^ flies. Wilcoxon rank sum tests are performed per gap width against respective pBDPGAL4U > *shi*^*ts*^ data (repeated from b in light green); *p < 0.05; **p < 0.01; ***p < 0.001. A detailed account of statistics is given in Supplementary Table 4.

Based on the ring-gap classification 60 R-lines > *shi*^*ts*^ were chosen for secondary high-speed video analyses at 200 frames/s and 34 °C (Fig. 2a). Lines were selected by strength and consistency of their phenotype; apparently blind and paralyzed lines were excluded. Their limits for the surmountable gap size and their behaviour at insurmountable gaps were determined by evaluating the percentage of approaches with climbing attempt and the percentage of successful crossings within 10 voluntary approaches to the gap per each of 10 or more flies per line and gap width. For one representative line per category Fig. 2b1–g1 shows final frames of 10-min recordings in the ring-gap assay (dead flies are marked), and Fig. 2b2–g2 the fractions of climbing trials, the percentage of falls into the gap and of successful transitions in the secondary assay. The 13 “very clumsy” lines either couldn’t shin up the climbing block consistently due to severe walking problems or they always fell into the gap. We therefore show a second “clumsy” example close to the “very clumsy” borderline. Because not all data sets are distributed normally, data are shown as box plots over gap width. As before, pBDPGAL4U > *shi*^*ts*^ (Fig. 2b and repeated as light green boxplots in 2c-g) served as control at 34 °C. The smallest evaluated gap size is 2.5 mm because flies start using climbing behaviour at this width. The median rate of climbing attempts at 2.5 mm in the controls is 80% and their success rate 70%. On the demanding end of 4.0 mm gap width the best individual control flies exhibited a 10%-probability to succeed but the median value is at 0%. The tests at 5.0 and 6.0-mm-wide, thus insurmountable gaps serve as control for correct width-estimation and decision-making capabilities. Indeed, the attempt rate of controls falls about linearly with increasing gap size. Figure 2c,d represent two underperforming lines with an interesting distinction. Flies of R61H03 > UAS-*shi*^*ts*^ (Fig. 2c; “overcautious”) are able to surmount 2.5 mm wide gaps but their overall rate of climbing attempts is very low. No fly fell into the gap; all walked normally when approaching the gap. Since almost each of their few trials was successful, their defect is to be seen in the sensory or motivational realm. In contrast, R24D10 > UAS-*shi*^*ts*^ flies (Fig. 2d) represent a class of “clumsy” flies close to a “very clumsy” rating in which the neuronal block causes problems already with walking. Half of the climbing attempts ended with the fly falling into the gap (Video 4). Their primary defect is therefore seen in a block within their motor system. In the line R23D06 > UAS-*shi*^*ts*^ (Fig. 2e; “clumsy”) the rate of trials reaches control level, whereas the success rate is significantly reduced due to falling particularly into the wide but surmountable gaps. The video sequences revealed a problem of these flies in attaching the foreleg tarsi to the distal side surface of the gap. Flies rather just pressed their bent tarsi against this wall. The markedly reduced grip results in slipping and falling in 13% of the attempts (Video 5). R22F08 > UAS-*shi*^*ts*^ represents an “overeager” phenotype (Fig. 2f). The rate of climbing initiation is increased at insurmountably broad gaps, but the relative success rate remains unchanged (Video 6). Flies can walk normally and in-depth analysis of their phenotype is laid out in the next section. Further, R68A11 > UAS-*shi*^*ts*^ exemplifies the “super-climber” category of hits (Fig. 2g; Video 7). At the just surmountable gap width of 4.0 mm this line has a significantly higher median chance of 10% of reaching the other side (40 transitions in 300 approaches) as compared to a 0% median chance of the control line (18 transitions in 440 approaches; p = 0.0021). Moreover, the “super-climber” line shows also more frequent attempts at insurmountable gaps. The flies are not physically larger than the control flies as is shown by comparing foreleg lengths (Fig. 2h; n = 23 “super climber” > UAS-*shi*^*ts*^; n = 24 pBDPGAL4U > UAS-*shi*^*ts*^; p = 0.425). Leg length is proportional to body size^14^ and a meaningful parameter for the physical climbing ability of a fly. Equal average body size most likely also holds true for the other R-lines used here as all have the same genetic background and were brought up with functioning nervous system.

### Mapping of an Overeager Phenotype to C2 or C3 Medulla Feedback Neurons

What can make a fly perform more climbing attempts at an insurmountable gap? Some lines in the ring-gap assay were able to spend more time at the demanding 4.0 mm gap because they overcame the smaller gaps in the screen quicker than controls. But also in the single-gap assay many lines from the “super-climber” and “overeager” categories showed enhanced initiation at the clearly insurmountable widths. Thus, the decision process as such might be altered in these flies. Decision making is among the most prominent tasks of brains. Decisions are based on sensory information from outside and inside the body and sometimes on learnt information from earlier encounters with similar situations. In our context flies need to know the gap width, but also their own capabilities. Perhaps, many of the inactivation lines are “overcautious”, because they acutely perceive feedback from a sudden incapacity imposed on them by turning on *shi*^*ts*^ and therefore do not try to climb even small gaps. Other lines, like our “clumsy” example close to the “very clumsy” rating, seem not to take their sudden incapacity into account and fail critically. “Overcautious” and “overeager” phenotypes could also result if flies perceive gaps as larger or smaller, respectively, due to an altered perception of gap size.

**Figure 3:**
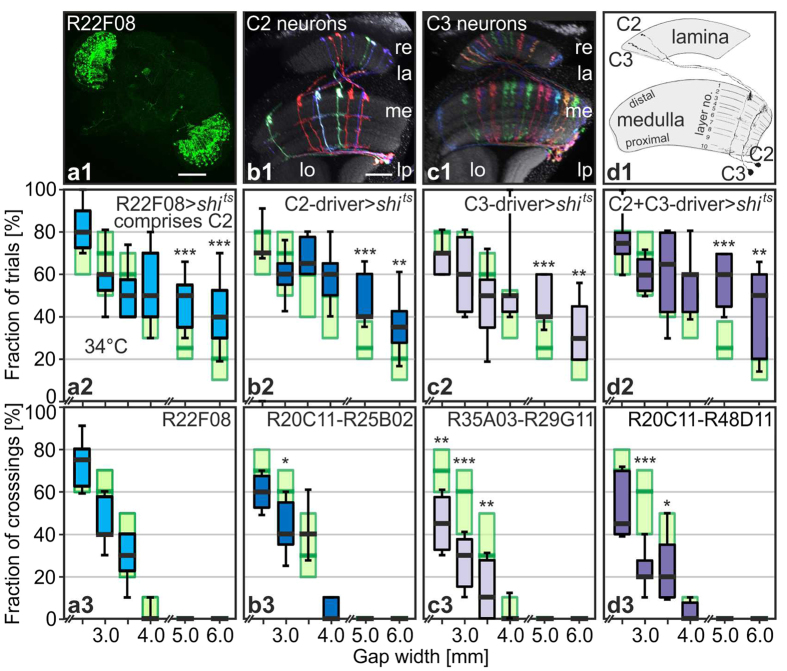
Overeager phenotype replicated by blocking just C2 and/or C3 feedback neurons. (**a1**) R22F08 expression pattern comprises scattered expression in C2 feedback neurons; scale bar, 100 μm. The split-GAL4 system is used to specifically target all C2 (**b**), all C3 neurons (**c**), or all C2 and C3 neurons (**d**). For visualization purposes, individual cells were labelled by the Multicolor FlpOut technique (**b1**,**c1**). Scale bar in b1, 20 μm, is valid for (**b1–d1**). (**d1**) Golgi reconstructions of C2 and C3 neurons in an outline of the optic lobes for comparison (modified after Fischbach and Dittrich, 1989)^15^. (**a2–d2**) fraction of climbing trials, and (**a3–d3**) fraction of successful crossings, respectively, both per 10 approaches to the gap per fly driving UAS-*shi*^*ts*^ at 34 °C. N = 10 or more flies per line and per two widths. Line-number combinations indicate AD-split GAL4 and DBD-split GAL4. Expression of *shi*^*ts*^ in all C2, all C3, or all C2 and C3 neurons leads to increased numbers of attempts at insurmountable gap widths. Wilcoxon rank sum tests against pBDPGAL4U > *shi*^*ts*^ control data (light green, taken from Fig. 3b) of the same gap width at 34 °C; *p < 0.05; **p < 0.01; ***p < 0.001. Panels a2 and a3 are reproduced from Fig. 2f2.

Flies determine gap width via the perception of the parallax motion created by structures on the distal side of the gap during approach^1^. Genetic analyses indicate that R1–6 but not R7 and R8 photoreceptors are required for gap crossing^1^ but other components of the relevant visual circuitry are unknown. Several of the “overeager” and “super-climber” lines show expression in the optic lobes (among other neuropils), suggesting that those phenotypes might be due to impaired gap-width estimation. To explore this possibility, we focused on the “overeager” example R22F08 > *shi*^*ts*^ introduced above. R22F08 has prominent yet patchy expression in the optic lobes but only limited expression elsewhere, primarily in one pair of antennal-lobe glomeruli and in about a dozen ventral-nerve-cord neurons^7^ (Fig. 3a1). C2 columnar feedback neurons projecting from the second visual neuropil, the medulla, to the most peripheral optic-lobe region, the lamina account for the optic-lobe expression^9^,^15^ (Fig. 3b1,d1). Only one other cell type, called C3, has similar columnar small-field projections from the medulla to the lamina, but C2 and C3 cells can be readily distinguished by the layers of arborisation in the medulla and the structure of their terminals in the lamina^9^,^15^ (Fig. 3c1,d1).

To examine a potential role of C2 neurons in gap-crossing behaviour, we made use of existing^9^ and newly generated split-GAL4 driver lines with highly specific expression in C2 neurons (Fig. 3b). Replication of the climbing experiments with [R20C11-R25B02] > *shi*^*ts*^ successfully reproduced the “overeager” phenotype (Fig. 3b2 shows proportion of approaches with trials and 3b3 proportion of successful approaches) and this result was further confirmed by testing two other C2-specific split combinations [R25B02-R48D11] and [R17C06-R25B02] (Supplementary Fig. S2). These results show that inactivation of C2 cells increases the number of climbing attempts at gaps of insurmountable width.

C2 and C3 cells have a similar overall structure^9^,^15^ (Fig. 3d1), overlapping sets of pre- and postsynaptic partners^16^,^17^,^18^ and are both thought to use GABA as their neurotransmitter^19^,^20^,^21^,^22^. We therefore tested the effect of silencing C3 neurons, again using specific split-Gal4 drivers. Inactivation with [R35A03-R29G11] > UAS-*shi*^*ts*^ (Fig. 3c2) or [R26H02-R29G11] > UAS-*shi*^*ts*^ (Supplementary Fig. S2) revealed no difference to the three C2 experiments. Finally we wanted to know whether the effects of C2 and C3 blockade are additive. Line [R20C11-R48D11] drives both cell types specifically; the effect of blocking on climbing initiation at the insurmountable gaps is significantly stronger than in the previous experiments but just at the 5.0-mm-wide gaps (Fig. 3d2). Together these results identify a role for two populations of columnar GABAergic feedback neurons of the early visual system, C2 and C3 cells, in gap-crossing behaviour.

### Activation of C2 or C3 Neurons Instead of Inactivation Turns Overeager into Overcautious Flies

**Figure 4:**
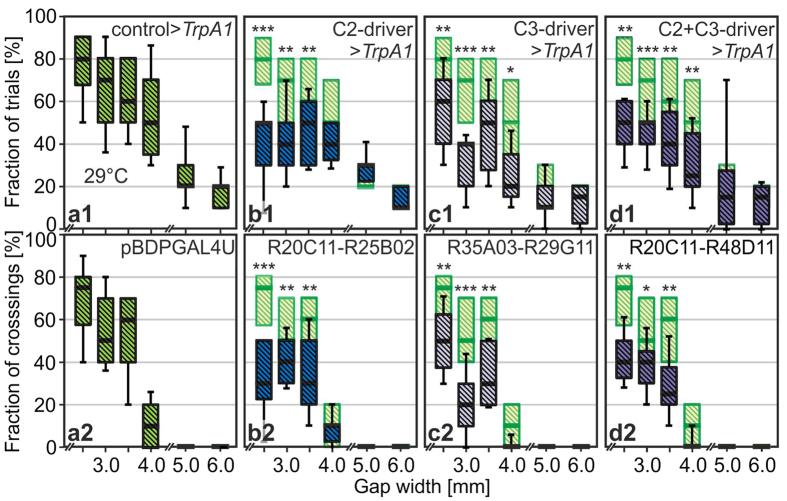
The overeager phenotype turns into an overcautious phenotype when activating instead of inactivating all C2, C3 or C2 and C3 feedback neurons. (**a**) Control line driving UAS-*dTrpA1* at 29 °C. (**b–d**) Lines and conventions as for Fig. 3 but lines are driving UAS-*dTrpA1* at 29 °C. N ≥ 10 flies or more per line and per two widths. Wilcoxon rank sum tests are performed per gap width against pBDPGAL4U > *dTrpA1* data obtained at 29 °C (light green, repeated from A); *p < 0.05; **p < 0.01; ***p < 0.001.

Blind or motion-blind flies hardly undertake any climbing attempts^1^ regardless of the gap width. By contrast, flies with blocked synaptic transmission of C2 and/or C3 neurons at large initiate climbing attempts more frequently than controls but their attempt rates nevertheless drop with increasing gap width. Thus, they can still perform some gap-width estimation, but silencing of those GABAergic cells might have caused an increased perception of parallax motion which would consequently make the distal wall of the gap appear closer. In this scenario, flies would make decisions consistently based on what they actually perceive. Would *activation* of C2 neurons then make the gaps appear wider? To test this hypothesis we crossed the C2 line [R20C11-R25B02] to UAS-*dTrpA1* so that the neurons could be artificially depolarized when shifting the temperature from 21 °C to 29 °C. Indeed, we find a significant reduction in the fraction of attempts at all surmountable gap widths from 2.5 to 4.0 mm in comparison to the control flies pBDPGAL4U > *dTrpA1* at 29 °C (Fig. 4a1,b1; controls repeated in light green for comparison). Likewise, the success rate went down (Fig. 4a2,b2). Replications of this activation experiment with two alternative C2-driver combinations yielded similar results (Supplementary Fig. S3). Statistically indistinguishable effects of turning “overeager” flies after C2-blocking into “overcautious” flies after C2-activation can consistently be reproduced when activating C3 neurons or C2 plus C3 neurons together (Fig. 4c,d). Replication with an alternative C3-driver combination is given in Supplementary Fig. S3. *dTrpA1* channels can be gradually activated depending on temperature^23^. We therefore tested the rates of climbing initiation and success of the C2 neuron line [R20C11-R25B02] > UAS-*dTrpA1* also at 27 °C instead of 29 °C (Supplementary Fig. S4). Indeed, an intermediate “overcautious” state can be seen.

### Reduction or Enhancement of Gap-Size Visibility Counters the Effects of C2/C3-Neuron Manipulation

**Figure 5:**
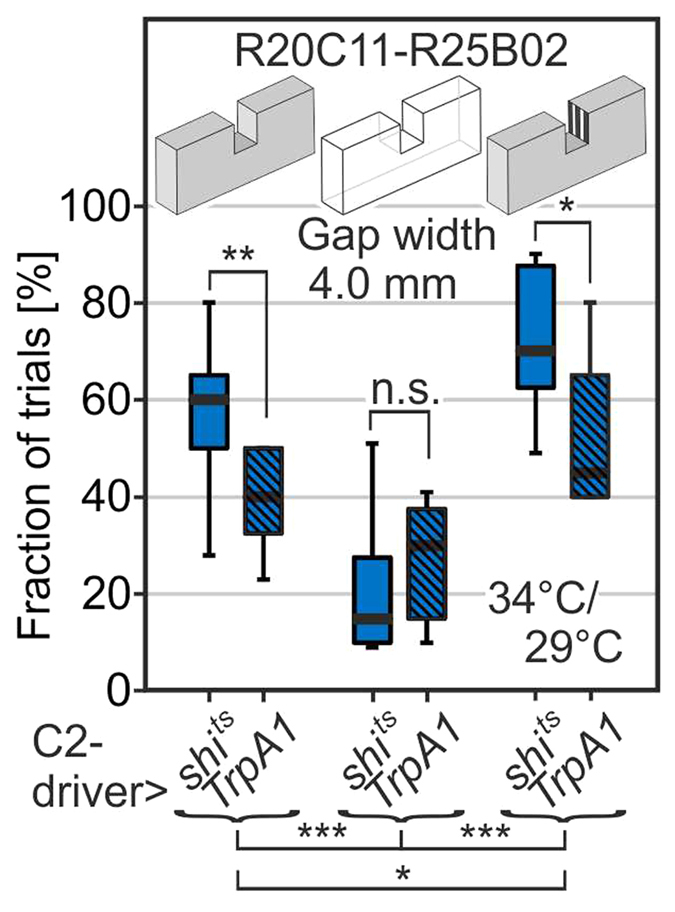
Overcautious or overeager behaviour can be elicited by visual properties of the gap’s opposite sidewall. Catwalks were either made of dark plastic material (as for all other experiments in this paper), clear Perspex, or dark plastic material and black and white vertical stripes at the opposite sidewall. Gap width was invariably 4.0 mm. Trials are most significantly reduced at the clear gap and highly significantly increased at the decorated gap. Neither blocking (*shi*^*ts*^ at 34 °C) nor activation (*dTrpA1* at 29 °C) of C2 neurons using (R20C11 AD-split GAL4 – R25B02 DBD-split GAL4) are effective when visibility of the opposite sidewall is low as in the case of the clear catwalk. If visibility is given C2-neuron blocking significantly increases the fraction of climbing trials as compared to C2-neuron activation.

The above results are consistent with the idea that flies overly initiate climbing at wide gaps because they actually *perceive* this gap as being smaller (C2/C3 blockade) and that they turn away from surmountable gaps because they actually *perceive* them as being wider (C2/C3 activation). The visual input informing the decision process, rather than the decision process itself, appears to have been altered. To test this hypothesis, we compensated for the C2/C3 phenotypes by modifying parallax-motion stimuli coming from the gaps. Reduction of visual motion-detector stimulation was achieved by testing flies on a clear Perspex catwalk. The translucent distal side offered just weak visual contrast in relation to the background. Enhancement of motion-detector stimulation was achieved by decorating the distal sidewall of a gap in a solid dark catwalk with vertical dark and bright stripes. This measure had previously increased the frequency of climbing attempts in wild-type flies^1^. In these experiments, we used the C2-driver line [R20C11-R25B02] to either drive UAS-*shi*^*ts*^ for inactivation (at 34 °C) or UAS-*dTrpA1* for activation (at 29 °C; Fig. 5). Consistent with the above interpretation of the C2/C3 phenotypes, experiments on the clear climbing block show that, if visual stimulation by the distal side is greatly reduced, climbing initiation is low and can neither be significantly boosted nor reduced by manipulating C2 neurons. However, if the distal side can be perceived well (dark block), or even perceived in an enhanced version (with black and white stripes), the flies initiate either more or fewer climbing attempts consistent with the enhancement or repression of parallax-motion perception by the inactivation or activation of the inhibitory C2 neurons, respectively.

## Discussion

We report the results of a large-scale genetic inactivation screen for neuronal populations with roles in gap-crossing behaviour. This screen identified sets of phenotypes providing entry points to the study of the neuronal basis of visual processing, decision making and motor control. Then we demonstrate a specific role of two related feedback-neuron types of the peripheral visual system in gap-width estimation.

The expression patterns of all GAL4-lines used in the screen are characterized in detail and neuroanatomical information can be downloaded^7^ (http://flweb.janelia.org/cgi-bin/flew.cgi). In combination with these expression data, our listed lines with aberrant climbing behaviour when driving *shi*^*ts*^ offer the opportunity to find novel regulatory centres in the central fly brain as well as components of sensory and motor pathways involved in climbing control. Expression patterns of the majority of these lines are not confined to a particular neuropil or neuron type; mapping of phenotypes therefore requires follow-up analysis. If expression is found in more than one location, usually just one or few will contribute to the behaviour under study. Moreover, seemingly similar expression in a particular neuropil like the central complex will in one line affect and in another line not affect the behaviour under study, because some but not all of the neuronal cell types there are involved in climbing behaviour. Conversely, a behavioural phenotype common to several lines at the level of resolution of the primary screen is not necessarily caused by expression in one common location or cell type. Primary phenotypes like falling into the grooves can be elicited, though with different behavioural fine-structure, in different ways involving different locations of the nervous system. Strong (motor) defects can obscure milder defects (like decision-making phenotypes) that will then go undetected. In summary, immediately meaningful correlations between behavioural phenotypes and expression patterns would require perfect resolution on the behavioural and the neuroanatomical side.

In spite of these challenges, we demonstrate here that combining analyses of the screen data with targeted follow-up experiments provides a general strategy to map individual cell types involved in gap crossing. The comparatively sparse expression of R-lines^7^ facilitates identification of candidate cell types and the modular nature of R-line transgenes provides the opportunity to refine expression to single cell types by employing the split-GAL4 technique^24^,^25^. As an example, we demonstrate the mapping of an “overeager” phenotype to specific C2/C3 visual interneurons. The same approach is applicable to all other categories of the primary screen, either by using split-GAL4 collections^9^,^26^, or by newly generating split-GAL4 drivers for candidate cell types and brain regions suggested by the screen results.

Consistent with the complex decision-making and motor-control requirements of gap climbing, the screen revealed a range of different defects which we scrutinized in high-speed video analyses in 60 cases. *Motor control* problems can either confine flies to a certain gap width or lead to an increased death rate if flies act without taking their acute limitations by the *shi*^*ts*^ neuronal block into account. In our “clumsy” example particularly control over the tarsi gets lost at the inactivation temperature. R23D06 > *shi*^*ts*^ flies were no longer able to attach their forelegs to the distal sidewall. Rather, they pressed their forelegs against the wall, gained some stability and dared to climb. But their reduced grip led to failures already at moderate, 2.5-mm-wide gaps and the overall death rate was high. A more severe motor-control defect was identified in the presented “clumsy” example close to the “very clumsy” borderline. Legs of walking R24D10 > *shi*^*ts*^ flies were uncoordinated, partially stiff and all flies were falling into the grooves. *Visual information* informs climbing behaviour at decision-making^1^ and execution stages^3^. As an example for the latter, several R-lines climbed with scattered azimuth orientation^3^ after inactivation. Certain other phenotypes can be caused either by interruptions in early visual-processing stages in the optic lobes or in *decision-making* stages in the central brain. Blind flies never climb and never show climbing attempts^1^ but by normal stepping they are expected to surmount sometimes 2.0-mm- and rarely 2.5-mm-wide gaps. The “overcautious” flies R61H03 > *shi*^*ts*^ surmounted 2.0 and 2.5 mm by climbing behaviour and basically stayed away from the 3.0-mm-wide gaps; all flies survived. This has been confirmed at the single gap under high-speed video surveillance. Since most of the rare climbing trials at 2.5-to-3.5-mm-wide gaps were again successful we surmise that decision making is negatively affected in this line. Note that the rates of attempts in R-lines are generally higher than the values published for WT Berlin. The latter were tested at room temperature^1^,^3^, but R-lines at 34 °C, the inactivation temperature for *shi*^*ts*^.

An unexpected outcome of the primary screen is the high number of “super-climber” lines in an inactivation screen. Differences in fly size are not expected since driver lines had the identical genetic background and were raised with functioning nervous system. Direct measurements of leg length as an indicator of body size^14^ confirmed that animals of selected lines were not bigger than controls. We tried to find adaptations that might enable a fly with an impaired nervous system to perform better than the healthy genetic background but did not observe obvious changes in the general crossing strategy of super climbers. Particularly, they did not jump over the gaps. Hypothetically, flies might overstretch their leg joints in an unhealthy way after an inhibition has fallen away by activating *shi*^*ts*^, but the most parsimonious explanation comes from comparison with the control experiments. The medians of the repetitions for each “super-climber” or “overeager” line signifying the climbing performance of 15 flies per experiment resides in most cases within the upper end of the normal range of the control flies (75% to 90% of pBDPGAL4U > *shi*^*ts*^). Many “super-climber” or “overeager” phenotypes are therefore not to be seen as an extension of the normal range but rather based on a consistently high trial rate at broader gaps. Indeed, kinematic data revealed that in the ring-gap assay many of those lines move out more quickly than controls and thus have more time left to try surmounting the widest grooves. This high trial rate ultimately leads to more success in the case of many “super climbers” and more casualties in the case of many “overeager” lines. However, some of these phenotypes are not the indirect result of faster crossing of smaller, surmountable gaps, because increased climbing persisted at the single gaps under high-speed-camera surveillance. Our characterization of the “overeager” line R22F08 > *shi*^*ts*^ demonstrates that altered visual perception can shift gap-crossing decisions so that these no longer match the actual gap width.

Decisions on climbing initiation are based on visual gap-size estimation. The amount of parallax motion is the decisive stimulus^1^, as in choice situations between several similar objects at different distances^2^. Visual feedback from *straight* locomotion signals relative proximity to an object when the motion of its retinal image is swift and over a wider angular range, but relative distance when the motion of the object image is slow and within a small angular range. In addition, retinal images of objects will expand as one gets closer (looming). In virtual-reality experiments either looming or parallax motion was taken out from the percept of flies in a choice situation between uniform objects at different distances^2^. The flies’ choices for the closer object were solely based on parallax motion and not on looming. Note that visual feedback from *rotation* does not carry distance information because all retinal images rotate at the same speed regardless of object distance. We surmise that the flies with artificially activated or inactivated C2 and C3 neurons in our present study take correct climbing decisions for the neurogenetically manipulated signals they perceive. The interpretation is backed up by the changes in climbing initiation found after manipulation of the gap’s visibility. Parallax motion is created by contrast edges of the opposite walls. If their visibility is greatly reduced by using clear Perspex material for the catwalk, climbing initiation is low, regardless of blocking or activating C2 neurons. A weak motion stimulus stays weak even after disinhibition and the distal wall seems far away for the fly. By contrast, decorating the opposite side with black and white vertical stripes enhances parallax motion^1^; the target side appears closer for the flies. Blocking of inhibition in this situation enhances the strong stimulus further and activation of inhibition reduces this strong stimulus. Decision making of the flies consistently follows the assumed strength of the stimulus reported by the visual system. In this model, the “overeager”/“overcautious” phenotypes of all C2 and/or C3 drivers tested are the results of correct decisions based on manipulated visual parallax-motion inputs to the decision stage. The most likely explanation is that we have manipulated the percept of the flies. The findings identify C2 and C3 cells as components of the neural circuits that perform gap-width estimation by parallax-motion processing.

In *Drosophila* C2 and C3 neurons were first described by Fischbach and Dittrich^15^ in their seminal Golgi analysis of the optic lobes. They are feedback neurons in the early visual system that project from the medulla, the second visual neuropil, outward to the lamina, the most peripheral optic-lobe region. Recent studies have added details of their light-level anatomy^9^ and their synaptic connections to other cells in the visual system^16^,^17^,^18^. C2 and C3 may be part of a negative feedback loop to the lamina^15^, although both cell types also have presynaptic sites in the medulla^16^. In addition to anatomy, the proposed role of C2/C3 as negative feedback neurons is based on immunohistochemical studies in *Drosophila* and other Diptera that indicate that both use GABA as their neurotransmitter^19^,^20^,^21^,^22^.

UAS-*shi*^*ts*^ block of C2 or C3 neurons led flies to initiate significantly more attempts at barely surmountable and insurmountable gaps whereas artificial depolarization of these cells with UAS-*dTrpA1* resulted in fewer attempts at clearly surmountable gaps. The *dTrpA1* effect was gradual with temperature (27 °C, 29 °C) as is the open-state probability of the underlying channels^10^,^23^. Blocking of C2 and C3 neurons together had some additive effect on the initiation of climbing at broad gaps. These findings are in accord with the idea that the perceived parallax motion is enhanced by inhibition of inhibitory C2/C3 neurons, or attenuated by artificially driving these neurons, respectively. Enhanced parallax motion will make the distal gap side appear closer than it actually is which boosts the readiness to climb. Reduced parallax motion, on the other hand, will make the landing side appear further away and consequently reduce the readiness to climb.

Connectivity data from EM reconstructions show synaptic interactions of C2/C3 cells with, among others, L1 and L2 lamina neurons^16^,^17^,^18^, which provide critical input to the fly’s motion-vision circuitry^9^,^27^,^28^,^29^,^30^. Earlier experiments with flying flies also indicated that C2/C3 cells play some role in motion vision^9^. Though the synaptic connections of C2 and C3 cells are distinct, the two cell types have several pre- and postsynaptic partners in common, in agreement with their similar phenotypes in this study.

The climbing success of flies with blocked C3 neurons (C3 > *shi*^*ts*^ and C2+C3 > *shi*^*ts*^ at 34 °C) was lower than that of flies with blocked C2 neurons (C2 > *shi*^*ts*^ at 34 °C). In six different test pairings between C2 > *shi*^*ts*^ and C3 > *shi*^*ts*^ flies at the surmountable gaps of 2.5, 3.0, 3.5, and 4.0 mm the C3 > *shi*^*ts*^ flies were significantly or highly significantly less successful in 11 out of 24 experiments and most non-significant comparisons had a bias towards a lower success rate of C3 > *shi*^*ts*^ flies. In three out of 12 test combinations between C2 > *shi*^*ts*^ and C2+C3 > *shi*^*ts*^ flies the latter were significantly less successful. By contrast, the success rate of C3 > *shi*^*ts*^ flies was statistically not different from that of C2+C3 > *shi*^*ts*^ flies (8 test combinations; Supplementary Table 5). The difference indicates specific functions of C2 and C3 neurons.

A possible clue to C2/C3 function is their apparent role as feedback neurons. For efficient neural coding, it is important to match the range of neuronal responses to the distribution of the expected input signals^31^. Appropriate neuronal adjustments might for example serve as gearshift between the mode of walking or flying which are associated with respective lower or higher visual-motion speeds. Modulation in sensitivity to visual motion in walking vs. standing flies has indeed been shown^32^ and a role in fine-tuning visual responses has been reported for another medulla-to-lamina feedback-neuron type, lamina wide-field neurons^33^. In contrast to wide-field neurons, C2 and C3 neurons are each present as one cell in each of ~750 visual columns, i.e. they can operate locally. This could be important for the processing of distance information, which needs to be computed by comparing local differences in visual motion.

The C2/C3 neurons may also play a role as matched filters in decision making. By the action of C2/C3 feedback neurons hungry or otherwise needy flies might actually perceive a gap as being smaller and objects as closer whereas saturated or generally wantless flies might perceive the same gap as being wider and objects as further away. Modifications of sensory information at the input stage have been described for the olfactory system in humans^34^ and flies^35^. In *Drosophila* local signalling by short neuropeptide F (sNPF) and a global metabolic insulin signal are integrated at odorant-receptor neurons expressing the olfactory-receptor type OR42b to modulate olfactory sensitivity. Starvation increases presynaptic activity via intraglomerular sNPF signaling^35^ and presynaptic facilitation in Or42b neurons is sufficient to mimic starvation-like behaviour in saturated flies. Starvation increases expression of the sNPF receptor in these neurons, thus changes the odour map and leads to robust food search behaviour. Similar processes involving C2/C3 might shape the visual percept of the world according to the needs of the animal.

## Methods

### Flies

R-lines from the library^6^ generated at HHMI Janelia Research Campus are inserted in the same genetic background and at the same genomic location, minimizing expression-independent differences between lines. For the primary screen R-GAL4 lines were blindly chosen and crossed to UAS-*shi*^*ts*^^8^ on Dextrose food (water, agar, dextrose, corn meal, yeast, Tegosept®, ethanol, proprionic acid) at 21.6 °C, 55% rel. humidity and a 16h/8h light/dark cycle. Two sets of incubators with an offset of 5h to each other were used and experiments run in time windows of 2h around the evening activity peak for the respective incubator. Three-to-four days before an experiment, three groups of 15 2-to-3-days old males were sorted out under cold anaesthesia (4 °C) and put on vials with Dextrose food. The three or more repetitions per line were spread over four-to-five days to average over a possible impact of fly age. An R-GAL4 line driving no expression (pBDPGAL4U) crossed to UAS-*shi*^*ts*^ was used as control for all experiments^6^. Split-GAL4 lines^24^ were generated as described^7^,^9^. AD-split GAL4 is on chromosome 2L (25D7) and DBD-split GAL4 is on 3L (68A4). They were crossed either to UAS-*shi*^*ts*^ or UAS-*dTrpA1*^10^. Just for high-speed video analyses, wings were shortened to 1/3 of their length under cold anaesthesia. Flies were given at least 12h to recover on food at 21.6 °C, 55% rel. humidity.

### Multicolor FlpOut (MCFO) anatomy

MCFO^36^ generates stochastically labelled single cells within the expression pattern of a driver line in a spectrum of colours. Briefly, UAS-reporter constructs carrying Flag, V5, and HA epitope tags downstream of an FRT-flanked stop signal were stochastically expressed by limited excision of the stop cassettes. MCFO-1 flies (pBPhsFlp2::PEST in attP3;; pJFRC201-10XUAS-FRT > STOP > FRT-myr::smGFP-HA in VK0005, pJFRC240-10XUAS-FRT > STOP > FRT-myr::smGFP-V5-THS-10XUAS-FRT > STOP > FRT-myr::smGFP-FLAG in su(Hw)attP1) were crossed to the driver lines of interest and female flies from these crosses processed and imaged as described^36^. The reoriented views shown in Fig. 1b1 were generated from confocal stacks using V3D^37^ and exported as screenshots.

### Ring-gap assay

The climbing disk (Ø 138 mm, height 15 mm) consists of mattfinished Perspex. Five concentric ring-gaps of 2.0-to-4.0-mm width (0.5 mm steps) and a bowl-like centre recess are milled into it. Figure 1a is drawn to scale. Ring-gaps are interconnected and filled half-high with high-salt water (2M; prevents evaporation) plus 0.5% detergent Triton-X with the help of a 50 ml syringe. Fifteen cold-anesthetized flies of a given line are placed into the recess and a glass plate is lowered onto the disk surface. This cover glass is lifted by 3.0 mm by a stepper motor when all flies are awake and the disk is vibrated for 10 s to activate the flies. The cover glass is coated with Sigmacote (Sigma; SL2) to prevent upside-down walking and the narrow ceiling effectively prevents flight in intact flies. Flies can distribute for 10 min; an overhead camera (Basler; A622f) connected to a PC with custom-made software records their whereabouts once every second. A red backlight (Advanced Illumination; BL0808-660) improves visibility of the flies in the videos. Design documents for the rig and software can be found at http://www.iorodeo.com. Custom-made software was written in Fiji^38^ to count dead flies at the end of the experiments and evaluate videos off-line. Parameters are mean maximum distance from centre, percentage of flies in grooves, walking activity, and time to reach the mean maximum distance from centre. Development of the mean distance from centre can be plotted over time.

### High-speed video assay

Two synchronized high-speed video cameras (Basler; A503k; lens Pentax, YF3528) take side and top views of a climbing fly^1^ at 200 frames/s (Fig. 2a1). Video information is fed to frame grabbers (Dalsa X64 Xcelera–CL PX4) and handled with custom-made software written in C# and using the Dalsa Sapera.NET library (Version 7.0). A single fly walks with shortened wings on a catwalk (30 mm long, 10 mm high and 4 mm wide) made of dark Delrin with a rectangular gap in the middle (Fig. 2a2). Gaps range from 2.0–to-6.0-mm width and are 5.0 mm deep. Ramps with a slope of 45° on both sides improve the number of voluntary visits on the catwalk, which is placed on a 50 mm diameter circular platform surrounded by water to prevent escape. The side view is taken against a white backlight (Smart Vision Lights; SOLB-150×150-WHI). The top view is illuminated by two overhead white LED spotlights (Mightex; SLS-0300-B). Each tested fly was observed for ten approaches to one gap size. The fraction of climbing initiations (indicated by “leg-over-head” behaviour)^1^, falls into the gap and fraction of successful transitions were noted. Means and s.e.m.s of these values for at least ten flies per line and gap size were determined. The ambient temperature for blocking experiments with UAS-*shi*^*ts*^ was 34 °C and 29 °C for activation experiments using UAS-*dTrpA1*. Qualitative observations indicating a possible explanation for failures were noted.

### Leg length

Forelegs were removed under anaesthesia and glued onto a microscope slide with double-sided adhesive tape and covered with clear shipping tape. A photo was taken under the microscope and measured using Fiji^38^. Five points were digitized and the lengths of femur, tibia, metatarsus, and the tarsi added.

## Additional Information

**How to cite this article**: Triphan, T. *et al*. A screen for constituents of motor control and decision making in *Drosophila* reveals visual distance-estimation neurons. *Sci. Rep.* **6**, 27000; doi: 10.1038/srep27000 (2016).

## Supplementary Material

Supplementary Information

Supplementary Video 1

Supplementary Video 2

Supplementary Video 3

Supplementary Video 4

Supplementary Video 5

Supplementary Video 6

Supplementary Video 7
