# Supplementary material for: A screen for constituents of motor control and decision making in Drosophila reveals visual distance-estimation neurons
Source: Sci Rep. 2016 Jun 3;6:27000. doi: 10.1038/srep27000 (PMC4891706; doi:10.1038/srep27000)
Supplement: Supplementary Information [file srep27000-s1.doc]

**Supplementary Information**

**Title: A screen for constituents of motor control and decision making in *Drosophila* reveals visual distance-estimation neurons**

**Authors:** Tilman Triphan^1,2^, Aljoscha Nern^1^, Sonia F. Roberts^1,3^, Wyatt Korff^1^, Daniel Q. Naiman^4^ and Roland Strauss^5^

**Affiliations:** 1) Janelia Research Campus, Howard Hughes Medical Institute,
 19700 Helix Drive, Ashburn, VA 20147, USA

2) Current address: Universität Konstanz, Zukunftskolleg, Universitätsstr. 10, 78464 Konstanz, Germany

3) Current address: University of Pennsylvania, Department of Electrical and Systems Engineering, 200 S. 33rd Street, Philadelphia, PA 19104, USA

4) Johns Hopkins University, Department of Applied Mathematics and Statistics, 3400 North Charles Street, Baltimore, MD 21218, USA

5) Johannes Gutenberg-Universität Mainz, Institut für Zoologie III, Col.-Kleinmann-Weg 2, 55099 Mainz, Germany

***) Corresponding Author:** RS (email: rstrauss@uni-mainz.de)

**
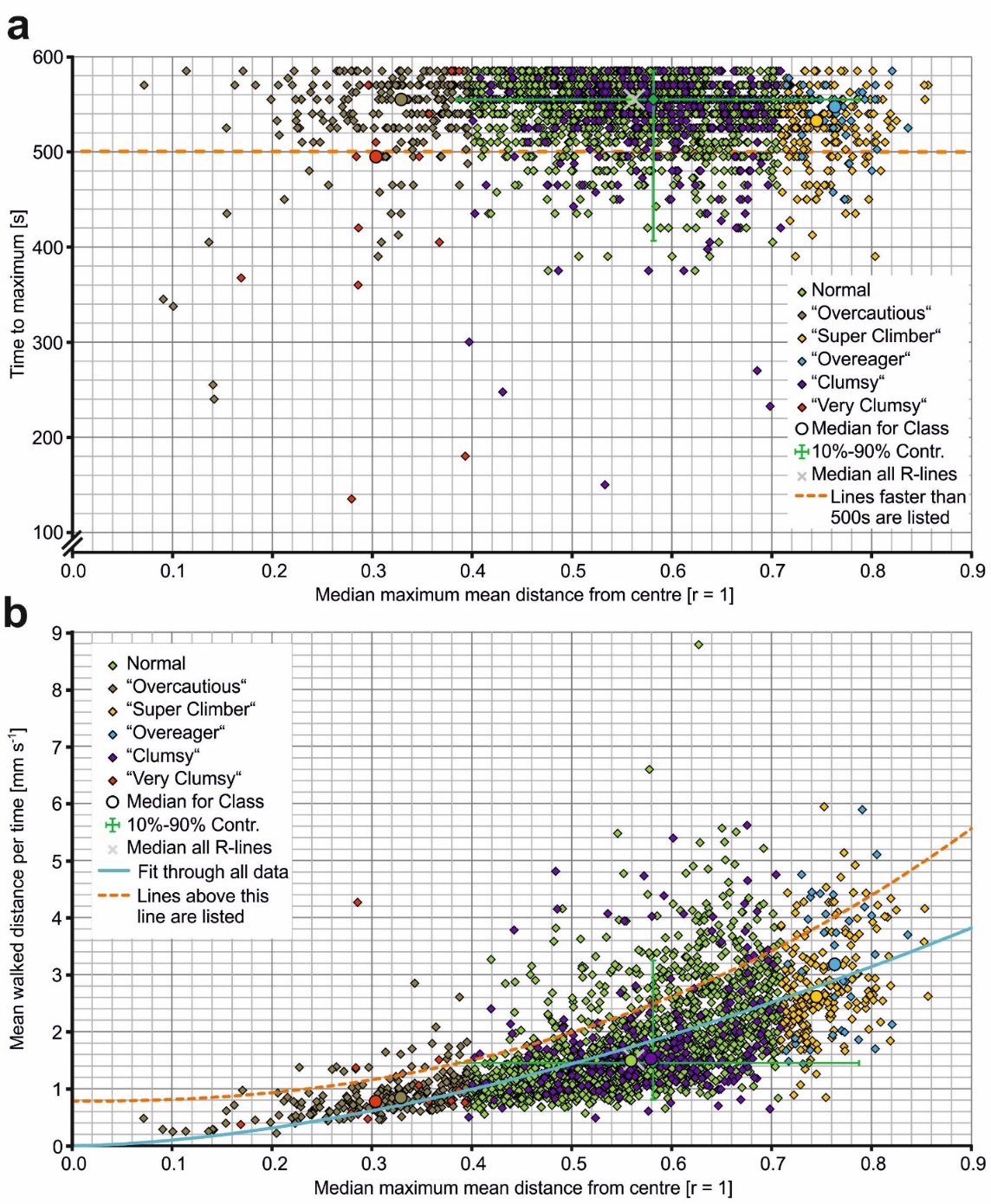
**

**Supplementary Fig. S1 Evaluation of the time taken to reach the maximum mean distance from centre and of walking activity for the ring-gap experiments of Figure 1 with 2,415 R-GAL4 lines driving UAS-*shibire^ts^* to inactivate sets of neurons at 34°C.** **a:** This evaluation picks up particularly fast-climbing lines. Diamonds represent 3 to 32 replications for a line with 15 flies each; the colours correspond to the classes from Fig. 1. The centre of the green cross represents the medians of 1,412 control experiments, each comprising 15 flies of pBDPGAL4U driving UAS-*shi^ts^* at 34°C, and the arms the 10%-/90%-quantiles. All lines that achieved their median maximum mean distance from centre in less than 500s are listed in **Supplementary Table 2**. **b:** Walking and climbing radially outward will create the least activity whereas swiftly walking alongside a circular groove without climbing can create the highest walking activity. Diamonds represent 3 to 32 replications for a line with 15 flies each; the colours correspond to the classes from Fig. 1. The centre of the green cross represents the medians of 1,412 control experiments, each comprising 15 flies of pBDPGAL4U driving UAS-*shi^ts^* at 34°C, and the arms the 10%-/90%-quantiles. All lines above a line x1,y1 = 0.12,0.0 and x2,y2 = 0.9,4.2 are listed in **Supplementary Table 3**.

**
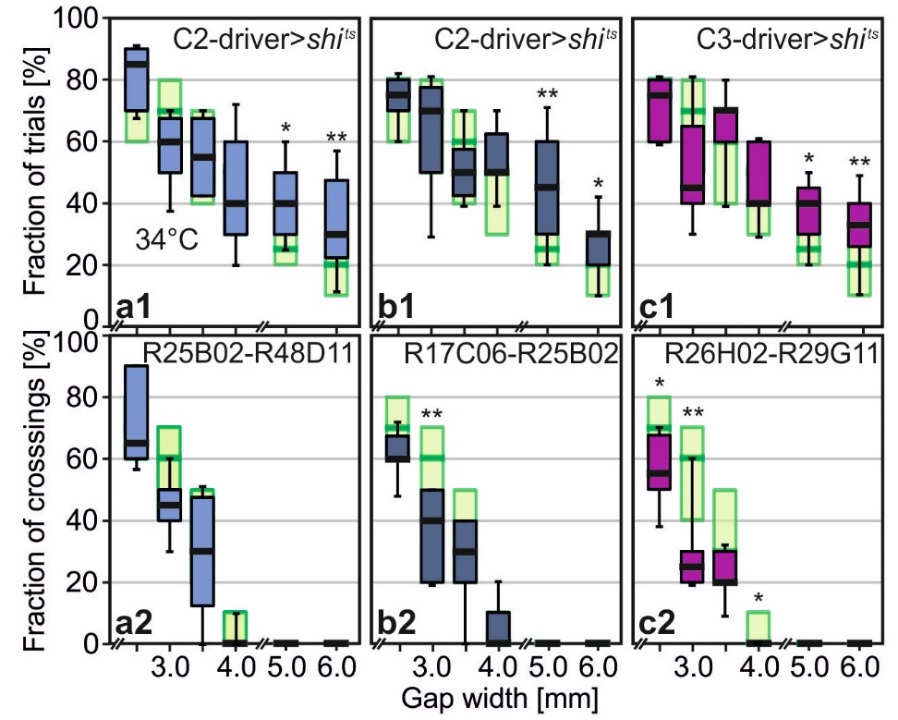
**

**Supplementary Fig. S2 Replication of the overeager phenotypes of Figure 3 using addit­ional split-GAL4 combinations for driving UAS-*shi^ts^ at 34°C*.** Conventions as for Fig. 3.

**
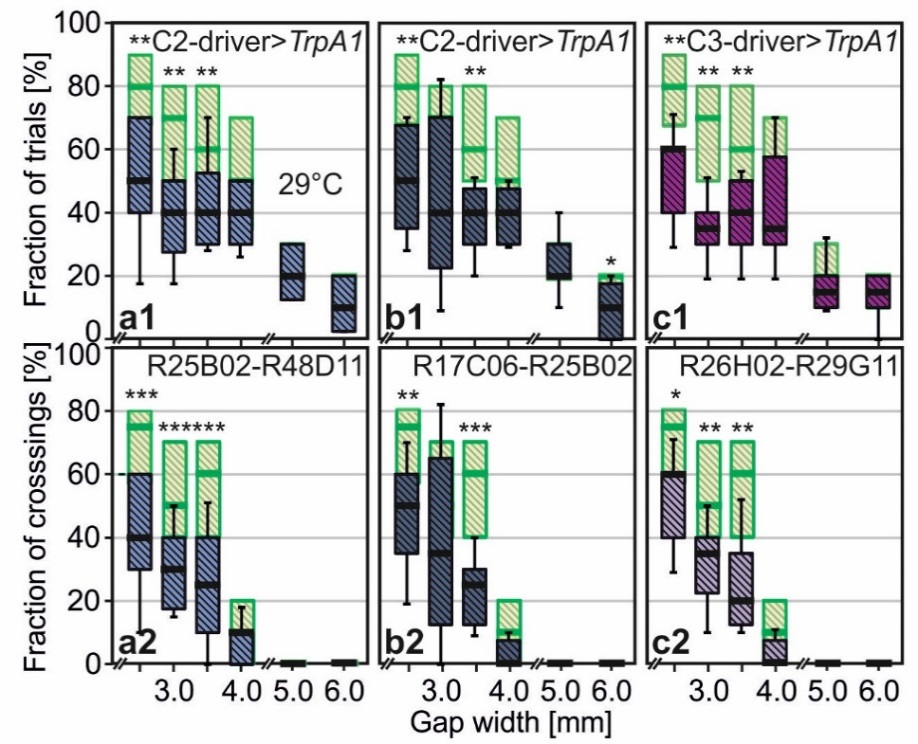
**

**Supplementary Fig. S3 Replication of the overcautious phenotypes of Figure 4 using the additional split-GAL4 combinations for driving UAS-*dTrpA1* at 29°C.** Conventions as for Fig. 4.

**
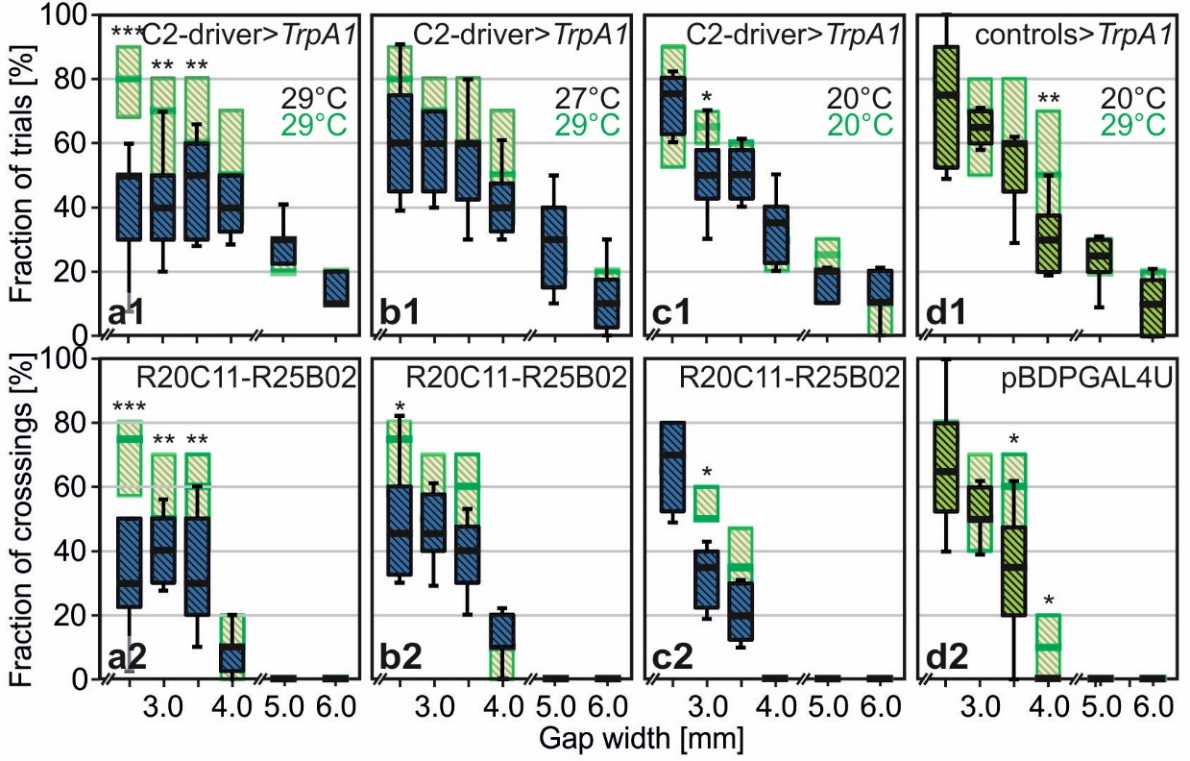
**

**Supplementary Fig. S4 Severity of the overcautious phenotype induced by *dTrpA1* expression in C2 neurons can be controlled by temperature.** The C2-specific [R20C11-R25B02] split-GAL4 driver combination is used to drive UAS-*dTrpA1* at 29°C (**a**) and 27°C (**b**). Panels a1 and a2 are reproduced from Fig. 4b1 and b2. An intermediate “overcautious” state can be seen at three of the six gap widths tested. All 29°C and 27°C data are statistically compared to pBDPGAL4U>*dTrpA1* control data obtained at 29°C (a, b). For comparison flies of the above combination and pBDPGAL4U control flies were both crossed to UAS-*dTrpA1* and tested at the restrictive temperature of 20°C (**c**); C2-flies behave normally with one exception at the 3 mm wide gap. The two control data sets are compared to each other (**d**, dark green 20°C, light green 29°C, taken from Fig. 4a). They show a moderate influence of temperature on climbing behaviour at 3.5 and 4.0 mm wide gaps. All tests are Wilcoxon rank sum tests.

**Supplementary Table 1 Line names of the hits in Figure 1 in five categories.** “Overcautious” and “very clumsy” lines underperform at normal and increased loss rates, respectively. “Clumsy” flies show normal climbing performance at an increased loss rate. “Overeager” and “super climber” flies perform better than controls at an increased and normal loss rate, respectively. n, number of repetitions (15 flies per repetition). MaxMeanDist, medians of the maximum mean distance from centre in relative units (0, centre; 1 outer rim of the climbing disk). % dead flies, median percentage of dead flies at the end of the 10-min experiments.

| **Overcautious** | | | | | | | | |
| --- | --- | --- | --- | --- | --- | --- | --- | --- |
| **Line name** | **n** | **MaxMeanDist** | **% dead flies** |  | **Line name** | **n** | **MaxMeanDist** | **% dead flies** |
| R10F06 | 4 | 0.391 ± 0.113 | 10.00 ± 14.40 |  | R35C09 | 6 | 0.341 ± 0.049 | 20.00 ± 11.20 |
| R10G02 | 3 | 0.348 ± 0.058 | 6.67 ± 4.00 |  | R36B07 | 3 | 0.334 ± 0.122 | 26.67 ± 17.60 |
| R10H09 | 3 | 0.309 ± 0.084 | 0.00 ± 11.50 |  | R37F05 | 3 | 0.382 ± 0.079 | 20.00 ± 16.80 |
| R11B03 | 6 | 0.349 ± 0.111 | 0.00 ± 2.90 |  | R37H01 | 7 | 0.370 ± 0.177 | 6.25 ± 9.40 |
| R11C05 | 9 | 0.272 ± 0.143 | 0.00 ± 2.20 |  | R38A05 | 3 | 0.356 ± 0.119 | 13.33 ± 10.20 |
| R11E07 | 3 | 0.240 ± 0.075 | 6.67 ± 3.90 |  | R38B09 | 3 | 0.387 ± 0.056 | 6.67 ± 34.60 |
| R11G10 | 3 | 0.310 ± 0.082 | 6.67 ± 4.00 |  | R38C10 | 6 | 0.389 ± 0.068 | 23.33 ± 15.00 |
| R12A12 | 3 | 0.345 ± 0.242 | 6.67 ± 3.90 |  | R38D03 | 5 | 0.309 ± 0.057 | 0.00 ± 3.00 |
| R12D02 | 9 | 0.377 ± 0.061 | 6.67 ± 7.00 |  | R38G04 | 3 | 0.212 ± 0.094 | 6.67 ± 3.90 |
| R12D04 | 3 | 0.272 ± 0.153 | 6.67 ± 4.00 |  | R38G07 | 9 | 0.276 ± 0.127 | 20.00 ± 22.20 |
| R12D05 | 6 | 0.364 ± 0.172 | 16.67 ± 27.40 |  | R38G08 | 6 | 0.346 ± 0.109 | 3.33 ± 20.90 |
| R12E09 | 3 | 0.284 ± 0.297 | 14.29 ± 13.70 |  | R38H05 | 3 | 0.154 ± 0.022 | 0.00 ± 3.90 |
| R12F01 | 6 | 0.395 ± 0.176 | 0.00 ± 5.40 |  | R39A03 | 3 | 0.386 ± 0.222 | 13.33 ± 13.90 |
| R12H11 | 6 | 0.380 ± 0.231 | 6.67 ± 6.20 |  | R39A05 | 6 | 0.325 ± 0.105 | 26.67 ± 25.60 |
| R13B07 | 7 | 0.331 ± 0.071 | 6.67 ± 5.60 |  | R39B03 | 3 | 0.336 ± 0.044 | 20.00 ± 7.70 |
| R13B10 | 6 | 0.379 ± 0.119 | 3.33 ± 15.60 |  | R39D12 | 3 | 0.348 ± 0.044 | 6.67 ± 6.70 |
| R13C09 | 7 | 0.322 ± 0.083 | 6.67 ± 7.30 |  | R39H01 | 5 | 0.245 ± 0.113 | 7.14 ± 6.70 |
| R13D09 | 3 | 0.155 ± 0.351 | 6.67 ± 6.70 |  | R40A01 | 6 | 0.362 ± 0.191 | 0.00 ± 5.60 |
| R13D11 | 7 | 0.380 ± 0.111 | 13.33 ± 12.80 |  | R40C07 | 8 | 0.318 ± 0.186 | 3.33 ± 11.80 |
| R13D12 | 5 | 0.265 ± 0.119 | 0.00 ± 5.90 |  | R40F09 | 3 | 0.310 ± 0.120 | 0.00 ± 8.30 |
| R13F09 | 6 | 0.393 ± 0.125 | 3.33 ± 12.90 |  | R42D11 | 6 | 0.216 ± 0.091 | 6.67 ± 3.50 |
| R13G06 | 6 | 0.365 ± 0.104 | 6.67 ± 10.00 |  | R42G10 | 6 | 0.272 ± 0.084 | 6.67 ± 16.00 |
| R14C08 | 6 | 0.375 ± 0.152 | 23.33 ± 23.30 |  | R43D09 | 9 | 0.367 ± 0.161 | 0.00 ± 7.40 |
| R14D11 | 6 | 0.309 ± 0.063 | 3.33 ± 6.50 |  | R45C10 | 6 | 0.358 ± 0.152 | 20.00 ± 24.70 |
| R14E06 | 9 | 0.243 ± 0.101 | 6.67 ± 6.50 |  | R45G06 | 10 | 0.276 ± 0.238 | 6.67 ± 9.50 |
| R14E12 | 3 | 0.343 ± 0.093 | 6.67 ± 4.00 |  | R46C04 | 6 | 0.375 ± 0.104 | 16.04 ± 16.70 |
| R14G03 | 3 | 0.384 ± 0.065 | 14.29 ± 21.20 |  | R46E11 | 6 | 0.380 ± 0.166 | 10.00 ± 5.40 |
| R14H02 | 9 | 0.321 ± 0.163 | 26.67 ± 12.10 |  | R47D06 | 6 | 0.395 ± 0.059 | 10.00 ± 10.20 |
| R15C05 | 3 | 0.389 ± 0.030 | 0.00 ± 0.00 |  | R47E02 | 3 | 0.291 ± 0.171 | 0.00 ± 3.90 |
| R15D10 | 3 | 0.278 ± 0.137 | 0.00 ± 3.90 |  | R47E07 | 27 | 0.220 ± 0.168 | 0.00 ± 7.80 |
| R15E01 | 6 | 0.304 ± 0.113 | 0.00 ± 5.90 |  | R47G10 | 9 | 0.359 ± 0.103 | 6.67 ± 12.50 |
| R15E11 | 3 | 0.072 ± 0.005 | 0.00 ± 0.00 |  | R48B04 | 3 | 0.206 ± 0.211 | 26.67 ± 10.20 |
| R16C03 | 3 | 0.369 ± 0.024 | 0.00 ± 7.70 |  | R48B12 | 3 | 0.396 ± 0.067 | 0.00 ± 0.00 |
| R16C06 | 8 | 0.280 ± 0.239 | 0.00 ± 3.10 |  | R48C07 | 3 | 0.369 ± 0.042 | 6.67 ± 3.80 |
| R16D02 | 6 | 0.355 ± 0.086 | 10.00 ± 20.70 |  | R48G04 | 3 | 0.199 ± 0.124 | 0.00 ± 0.00 |
| R16F08 | 5 | 0.327 ± 0.067 | 6.67 ± 12.10 |  | R49A06 | 3 | 0.327 ± 0.133 | 26.67 ± 11.50 |
| R16G04 | 9 | 0.316 ± 0.111 | 6.67 ± 21.80 |  | R49F10 | 3 | 0.240 ± 0.023 | 0.00 ± 0.00 |
| R17A11 | 8 | 0.388 ± 0.125 | 18.68 ± 16.30 |  | R49H02 | 6 | 0.336 ± 0.112 | 3.33 ± 3.70 |
| R17F03 | 8 | 0.368 ± 0.183 | 13.33 ± 10.50 |  | R49H05 | 6 | 0.258 ± 0.113 | 6.67 ± 5.00 |
| **Overcautious cont’** | | | | | | | | |
| **Line name** | **n** | **MaxMeanDist** | **% dead flies** |  | **Line name** | **n** | **MaxMeanDist** | **% dead flies** |
| R17G03 | 9 | 0.225 ± 0.171 | 0.00 ± 5.20 |  | R50B11 | 9 | 0.327 ± 0.111 | 7.14 ± 8.90 |
| R18F09 | 3 | 0.330 ± 0.160 | 0.00 ± 0.00 |  | R50E07 | 3 | 0.315 ± 0.032 | 14.29 ± 6.70 |
| R19A11 | 6 | 0.321 ± 0.125 | 3.33 ± 5.40 |  | R51B06 | 9 | 0.255 ± 0.101 | 6.67 ± 3.50 |
| R19B07 | 5 | 0.275 ± 0.282 | 0.00 ± 20.30 |  | R51C01 | 6 | 0.142 ± 0.020 | 0.00 ± 3.50 |
| R20C08 | 9 | 0.395 ± 0.100 | 20.00 ± 20.10 |  | R51G01 | 3 | 0.327 ± 0.119 | 6.67 ± 3.90 |
| R20D01 | 3 | 0.366 ± 0.096 | 13.33 ± 17.60 |  | R51H03 | 6 | 0.229 ± 0.150 | 0.00 ± 40.40 |
| R20F06 | 4 | 0.329 ± 0.249 | 3.57 ± 15.80 |  | R52B10 | 3 | 0.341 ± 0.061 | 13.33 ± 14.30 |
| R20G04 | 5 | 0.379 ± 0.317 | 13.33 ± 5.60 |  | R52C06 | 6 | 0.224 ± 0.040 | 0.00 ± 8.20 |
| R21A02 | 7 | 0.336 ± 0.137 | 0.00 ± 24.60 |  | R52D06 | 6 | 0.392 ± 0.203 | 0.00 ± 14.90 |
| R21G06 | 9 | 0.288 ± 0.161 | 0.00 ± 8.00 |  | R52G01 | 6 | 0.324 ± 0.075 | 6.67 ± 2.80 |
| R21G09 | 6 | 0.396 ± 0.200 | 6.25 ± 8.80 |  | R52H01 | 3 | 0.141 ± 0.014 | 0.00 ± 8.30 |
| R21H02 | 3 | 0.306 ± 0.023 | 0.00 ± 0.00 |  | R53B04 | 3 | 0.241 ± 0.281 | 6.67 ± 25.30 |
| R22B02 | 9 | 0.383 ± 0.246 | 6.67 ± 5.30 |  | R53D12 | 6 | 0.284 ± 0.052 | 0.00 ± 5.70 |
| R22C04 | 5 | 0.340 ± 0.358 | 0.00 ± 9.40 |  | R53E04 | 3 | 0.223 ± 0.043 | 0.00 ± 3.90 |
| R22F03 | 10 | 0.310 ± 0.291 | 3.33 ± 22.70 |  | R53F12 | 9 | 0.367 ± 0.099 | 6.67 ± 3.70 |
| R22F06 | 3 | 0.329 ± 0.079 | 13.33 ± 2.90 |  | R53G03 | 3 | 0.371 ± 0.065 | 6.67 ± 6.70 |
| R22G02 | 6 | 0.205 ± 0.057 | 16.67 ± 20.40 |  | R53G11 | 3 | 0.295 ± 0.092 | 0.00 ± 11.50 |
| R22G07 | 12 | 0.207 ± 0.173 | 0.00 ± 11.50 |  | R54A08 | 3 | 0.380 ± 0.071 | 6.67 ± 10.20 |
| R23A06 | 10 | 0.377 ± 0.119 | 6.67 ± 5.30 |  | R54B11 | 6 | 0.222 ± 0.168 | 3.33 ± 10.30 |
| R23G10 | 3 | 0.388 ± 0.171 | 0.00 ± 16.50 |  | R54H04 | 6 | 0.379 ± 0.113 | 0.00 ± 3.50 |
| R23G11 | 3 | 0.377 ± 0.064 | 26.67 ± 30.10 |  | R55A03 | 6 | 0.393 ± 0.157 | 3.33 ± 10.30 |
| R24A04 | 3 | 0.287 ± 0.043 | 6.67 ± 15.40 |  | R55A07 | 6 | 0.371 ± 0.216 | 13.33 ± 6.40 |
| R24B04 | 3 | 0.379 ± 0.142 | 20.00 ± 16.80 |  | R55D05 | 6 | 0.364 ± 0.049 | 10.00 ± 10.10 |
| R24C12 | 8 | 0.326 ± 0.203 | 10.00 ± 6.70 |  | R56A08 | 3 | 0.348 ± 0.152 | 20.00 ± 12.50 |
| R24D05 | 3 | 0.091 ± 0.020 | 0.00 ± 3.90 |  | R56A09 | 6 | 0.383 ± 0.138 | 10.00 ± 12.40 |
| R24F07 | 3 | 0.395 ± 0.103 | 0.00 ± 3.90 |  | R56H02 | 6 | 0.163 ± 0.100 | 0.00 ± 8.90 |
| R24F10 | 6 | 0.314 ± 0.135 | 14.05 ± 33.80 |  | R57C10 | 6 | 0.101 ± 0.045 | 0.00 ± 5.40 |
| R24H02 | 6 | 0.319 ± 0.075 | 0.00 ± 10.70 |  | R60E05 | 3 | 0.384 ± 0.170 | 6.67 ± 15.40 |
| R25A02 | 8 | 0.307 ± 0.132 | 3.33 ± 6.60 |  | R60G10 | 3 | 0.316 ± 0.136 | 6.67 ± 3.90 |
| R25A06 | 3 | 0.361 ± 0.033 | 6.67 ± 3.80 |  | R61H03 | 14 | 0.343 ± 0.150 | 3.33 ± 15.80 |
| R25A11 | 9 | 0.334 ± 0.188 | 6.67 ± 12.20 |  | R64B07 | 3 | 0.268 ± 0.060 | 0.00 ± 0.00 |
| R25B07 | 3 | 0.355 ± 0.158 | 28.57 ± 15.10 |  | R64D05 | 3 | 0.301 ± 0.133 | 0.00 ± 7.70 |
| R25C02 | 5 | 0.281 ± 0.046 | 0.00 ± 7.30 |  | R64F01 | 6 | 0.395 ± 0.154 | 20.00 ± 11.00 |
| R25D10 | 9 | 0.237 ± 0.230 | 0.00 ± 6.70 |  | R64G07 | 6 | 0.279 ± 0.057 | 6.67 ± 5.20 |
| R25F09 | 6 | 0.335 ± 0.100 | 0.00 ± 8.10 |  | R65B06 | 3 | 0.337 ± 0.173 | 0.00 ± 3.90 |
| R25H08 | 3 | 0.267 ± 0.117 | 6.67 ± 4.00 |  | R66B02 | 3 | 0.392 ± 0.092 | 26.67 ± 15.40 |
| R26B06 | 3 | 0.352 ± 0.058 | 20.00 ± 10.20 |  | R66B05 | 3 | 0.353 ± 0.208 | 13.33 ± 14.30 |
| R26B09 | 6 | 0.370 ± 0.111 | 3.33 ± 6.50 |  | R66B12 | 6 | 0.285 ± 0.229 | 3.33 ± 5.40 |
| R26B12 | 3 | 0.183 ± 0.038 | 0.00 ± 0.00 |  | R67B11 | 3 | 0.395 ± 0.079 | 13.33 ± 6.70 |
| R26D03 | 6 | 0.302 ± 0.115 | 0.00 ± 3.50 |  | R67D04 | 3 | 0.228 ± 0.011 | 20.00 ± 11.50 |
| R26D10 | 6 | 0.257 ± 0.130 | 0.00 ± 3.40 |  | R67E05 | 6 | 0.270 ± 0.081 | 0.00 ± 0.00 |
| R26E01 | 12 | 0.374 ± 0.137 | 13.33 ± 12.00 |  | R70B10 | 6 | 0.261 ± 0.066 | 6.67 ± 6.90 |
| R26E08 | 4 | 0.366 ± 0.129 | 6.67 ± 36.00 |  | R70F05 | 9 | 0.297 ± 0.073 | 0.00 ± 5.80 |
| R26F02 | 6 | 0.339 ± 0.046 | 3.33 ± 6.50 |  | R70G12 | 3 | 0.297 ± 0.133 | 13.33 ± 4.70 |
| R26F04 | 3 | 0.114 ± 0.013 | 0.00 ± 8.30 |  | R70H07 | 17 | 0.286 ± 0.089 | 13.33 ± 17.40 |
| R26F09 | 9 | 0.225 ± 0.050 | 0.00 ± 8.80 |  | R71C02 | 5 | 0.375 ± 0.105 | 13.33 ± 11.80 |
| R26H06 | 3 | 0.311 ± 0.058 | 0.00 ± 7.20 |  | R71F05 | 6 | 0.351 ± 0.196 | 3.33 ± 6.80 |
| R26H10 | 3 | 0.241 ± 0.102 | 7.14 ± 10.10 |  | R72E09 | 6 | 0.341 ± 0.245 | 13.33 ± 16.10 |
| R27B01 | 6 | 0.288 ± 0.087 | 6.67 ± 7.80 |  | R72F03 | 5 | 0.365 ± 0.106 | 0.00 ± 5.90 |
| R27E05 | 3 | 0.249 ± 0.077 | 0.00 ± 0.00 |  | R74F09 | 6 | 0.318 ± 0.092 | 3.33 ± 18.20 |
| R27E09 | 3 | 0.379 ± 0.079 | 0.00 ± 3.90 |  | R75H07 | 6 | 0.251 ± 0.066 | 3.33 ± 8.40 |
| R27H06 | 3 | 0.327 ± 0.225 | 6.67 ± 6.70 |  | R76H04 | 3 | 0.372 ± 0.229 | 13.33 ± 31.50 |
| R28A07 | 3 | 0.371 ± 0.152 | 13.33 ± 7.70 |  | R81A06 | 6 | 0.394 ± 0.157 | 0.00 ± 2.70 |
| R28B05 | 3 | 0.245 ± 0.148 | 0.00 ± 3.90 |  | R81B12 | 6 | 0.341 ± 0.163 | 3.12 ± 10.60 |
| R28C03 | 6 | 0.356 ± 0.163 | 6.67 ± 3.60 |  | R83A12 | 6 | 0.282 ± 0.101 | 3.12 ± 8.40 |
| R28F02 | 3 | 0.319 ± 0.094 | 0.00 ± 4.10 |  | R83B08 | 9 | 0.382 ± 0.084 | 0.00 ± 7.30 |
| R28H03 | 6 | 0.330 ± 0.108 | 6.67 ± 7.80 |  | R83H12 | 8 | 0.238 ± 0.081 | 0.00 ± 9.70 |
| R29D08 | 3 | 0.329 ± 0.139 | 13.33 ± 0.00 |  | R84C04 | 6 | 0.351 ± 0.159 | 20.95 ± 13.60 |
| R29G10 | 9 | 0.316 ± 0.115 | 0.00 ± 13.70 |  | R84E05 | 6 | 0.363 ± 0.141 | 6.67 ± 15.40 |
| R29H10 | 3 | 0.315 ± 0.150 | 0.00 ± 3.90 |  | R84H06 | 7 | 0.248 ± 0.062 | 15.38 ± 17.90 |
| **Overcautious cont’** | | | | | | | | |
| **Line name** | **n** | **MaxMeanDist** | **% dead flies** |  | **Line name** | **n** | **MaxMeanDist** | **% dead flies** |
| R30F10 | 6 | 0.395 ± 0.104 | 6.67 ± 6.20 |  | R86B11 | 6 | 0.357 ± 0.209 | 30.00 ± 30.30 |
| R30H08 | 3 | 0.265 ± 0.177 | 0.00 ± 7.70 |  | R87C02 | 6 | 0.361 ± 0.149 | 6.67 ± 21.80 |
| R31A05 | 9 | 0.340 ± 0.082 | 6.67 ± 7.00 |  | R87G07 | 6 | 0.364 ± 0.173 | 3.33 ± 3.70 |
| R31H09 | 6 | 0.357 ± 0.137 | 10.00 ± 9.50 |  | R88H01 | 6 | 0.310 ± 0.097 | 10.00 ± 12.10 |
| R32B12 | 3 | 0.316 ± 0.170 | 0.00 ± 8.30 |  | R91D09 | 6 | 0.137 ± 0.054 | 0.00 ± 3.50 |
| R32D05 | 3 | 0.377 ± 0.147 | 20.00 ± 20.40 |  | R92C05 | 3 | 0.320 ± 0.105 | 20.00 ± 7.70 |
| R32D06 | 6 | 0.396 ± 0.084 | 3.33 ± 20.00 |  | R92C11 | 3 | 0.346 ± 0.030 | 6.67 ± 7.70 |
| R32D11 | 6 | 0.370 ± 0.249 | 6.67 ± 11.00 |  | R92D09 | 6 | 0.308 ± 0.091 | 13.81 ± 6.30 |
| R32E01 | 3 | 0.338 ± 0.161 | 0.00 ± 34.60 |  | R93B10 | 9 | 0.230 ± 0.082 | 13.33 ± 8.40 |
| R33A12 | 6 | 0.280 ± 0.074 | 3.33 ± 5.80 |  | R93D10 | 6 | 0.395 ± 0.225 | 10.00 ± 3.60 |
| R33C06 | 6 | 0.256 ± 0.185 | 3.33 ± 8.40 |  | R93F03 | 3 | 0.329 ± 0.105 | 0.00 ± 0.00 |
| R34A03 | 3 | 0.388 ± 0.088 | 13.33 ± 7.70 |  | R93F07 | 6 | 0.345 ± 0.086 | 0.00 ± 5.60 |
| R34C03 | 3 | 0.171 ± 0.043 | 6.67 ± 0.20 |  | R93G02 | 6 | 0.324 ± 0.275 | 0.00 ± 13.60 |
| R35A12 | 3 | 0.376 ± 0.128 | 0.00 ± 0.00 |  | R93G05 | 3 | 0.306 ± 0.133 | 6.67 ± 7.20 |
| R35B10 | 3 | 0.313 ± 0.191 | 6.67 ± 13.90 |  | R93G12 | 3 | 0.382 ± 0.050 | 0.00 ± 3.90 |
| R35B11 | 5 | 0.229 ± 0.178 | 6.67 ± 3.70 |  |  |  |  |  |
| **Very Clumsy** | | | | | | | | |
| **Line name** | **n** | **MaxMeanDist** | **% dead flies** |  | **Line name** | **n** | **MaxMeanDist** | **% dead flies** |
| R11E12 | 6 | 0.393 ± 0.111 | 100.00 ± 3.90 |  | R41E06 | 6 | 0.304 ± 0.094 | 60.00 ± 30.00 |
| R16D07 | 3 | 0.280 ± 0.062 | 100.00 ± 0.00 |  | R59B03 | 6 | 0.347 ± 0.007 | 40.00 ± 1.70 |
| R16F05 | 3 | 0.284 ± 0.096 | 53.30 ± 31.50 |  | R71E05 | 3 | 0.296 ± 0.024 | 64.30 ± 7.30 |
| R17C08 | 7 | 0.367 ± 0.212 | 33.30 ± 19.20 |  | R75G02 | 3 | 0.286 ± 0.138 | 80.00 ± 6.90 |
| R17D03 | 3 | 0.169 ± 0.078 | 54.20 ± 39.70 |  | R84F07 | 6 | 0.357 ± 0.140 | 36.70 ± 41.50 |
| R34E11 | 4 | 0.286 ± 0.048 | 40.00 ± 16.80 |  | R92E06 | 4 | 0.387 ± 0.072 | 33.30 ± 0.00 |
| R38D10 | 3 | 0.380 ± 0.128 | 33.30 ± 16.00 |  |  |  |  |  |
| **Clumsy** | | | | | | | | |
| **Line name** | **n** | **MaxMeanDist** | **% dead flies** |  | **Line name** | **n** | **MaxMeanDist** | **% dead flies** |
| R09D03 | 5 | 0.457 ± 0.064 | 40.00 ± 22.40 |  | R41A07 | 9 | 0.488 ± 0.114 | 35.70 ± 25.40 |
| R10B03 | 3 | 0.612 ± 0.039 | 40.00 ± 11.30 |  | R41B01 | 6 | 0.506 ± 0.151 | 35.00 ± 21.30 |
| R10E04 | 9 | 0.676 ± 0.083 | 40.00 ± 21.40 |  | R41C12 | 3 | 0.641 ± 0.172 | 50.00 ± 26.80 |
| R10F07 | 3 | 0.654 ± 0.176 | 46.70 ± 17.60 |  | R41F05 | 3 | 0.590 ± 0.082 | 71.40 ± 41.80 |
| R10F11 | 6 | 0.483 ± 0.049 | 33.30 ± 26.30 |  | R41F12 | 6 | 0.561 ± 0.084 | 35.70 ± 24.20 |
| R11B04 | 6 | 0.475 ± 0.067 | 70.00 ± 31.10 |  | R41G05 | 3 | 0.473 ± 0.171 | 40.00 ± 7.70 |
| R12A02 | 6 | 0.664 ± 0.090 | 36.70 ± 26.50 |  | R41G08 | 15 | 0.599 ± 0.125 | 35.70 ± 33.00 |
| R12C02 | 3 | 0.484 ± 0.055 | 53.30 ± 25.30 |  | R42A06 | 6 | 0.510 ± 0.163 | 41.40 ± 26.60 |
| R12C11 | 5 | 0.475 ± 0.050 | 33.30 ± 14.00 |  | R42C01 | 9 | 0.705 ± 0.065 | 33.30 ± 19.20 |
| R13F10 | 3 | 0.691 ± 0.030 | 46.70 ± 23.40 |  | R42C09 | 6 | 0.551 ± 0.077 | 80.00 ± 30.60 |
| R14B01 | 3 | 0.609 ± 0.041 | 40.00 ± 17.70 |  | R42F03 | 3 | 0.533 ± 0.068 | 100.00 ± 10.50 |
| R14C12 | 6 | 0.613 ± 0.068 | 43.30 ± 31.80 |  | R42F05 | 3 | 0.618 ± 0.043 | 46.70 ± 19.30 |
| R14E08 | 3 | 0.554 ± 0.034 | 40.00 ± 7.70 |  | R42G08 | 3 | 0.623 ± 0.047 | 46.70 ± 7.70 |
| R14F12 | 6 | 0.639 ± 0.092 | 76.70 ± 26.10 |  | R42G12 | 3 | 0.573 ± 0.029 | 50.00 ± 10.00 |
| R14G02 | 6 | 0.586 ± 0.126 | 60.00 ± 28.50 |  | R42H08 | 6 | 0.615 ± 0.103 | 55.00 ± 39.90 |
| R14G08 | 6 | 0.515 ± 0.153 | 33.30 ± 15.80 |  | R43B12 | 6 | 0.600 ± 0.080 | 36.70 ± 19.00 |
| R14H05 | 9 | 0.483 ± 0.148 | 33.30 ± 28.50 |  | R43C10 | 6 | 0.564 ± 0.116 | 41.90 ± 32.70 |
| R16F02 | 6 | 0.420 ± 0.099 | 50.00 ± 23.60 |  | R43G08 | 3 | 0.509 ± 0.044 | 46.70 ± 27.70 |
| R16H03 | 5 | 0.577 ± 0.110 | 100.00 ± 38.80 |  | R44A01 | 9 | 0.652 ± 0.119 | 66.70 ± 32.20 |
| R17A10 | 6 | 0.531 ± 0.136 | 33.30 ± 27.30 |  | R44D05 | 3 | 0.457 ± 0.019 | 33.30 ± 21.40 |
| R17D06 | 6 | 0.492 ± 0.079 | 36.70 ± 29.60 |  | R45G05 | 6 | 0.573 ± 0.112 | 43.30 ± 20.40 |
| R17D09 | 6 | 0.555 ± 0.089 | 53.30 ± 38.80 |  | R46A06 | 6 | 0.609 ± 0.061 | 55.00 ± 17.70 |
| R17F04 | 3 | 0.683 ± 0.064 | 40.00 ± 25.30 |  | R47A12 | 6 | 0.521 ± 0.163 | 46.70 ± 21.20 |
| R17G01 | 5 | 0.501 ± 0.065 | 40.00 ± 27.40 |  | R47F01 | 6 | 0.613 ± 0.158 | 40.00 ± 24.60 |
| R17G05 | 9 | 0.559 ± 0.161 | 46.70 ± 27.70 |  | R47F08 | 6 | 0.523 ± 0.131 | 40.00 ± 6.00 |
| R19A06 | 4 | 0.518 ± 0.111 | 40.00 ± 18.00 |  | R47G04 | 6 | 0.506 ± 0.105 | 43.30 ± 16.70 |
| R19B03 | 6 | 0.502 ± 0.085 | 38.20 ± 30.60 |  | R47H11 | 6 | 0.536 ± 0.044 | 40.00 ± 33.50 |
| R19C07 | 6 | 0.466 ± 0.069 | 34.50 ± 26.40 |  | R48A07 | 6 | 0.500 ± 0.119 | 40.00 ± 16.70 |
| R19F02 | 6 | 0.576 ± 0.058 | 43.30 ± 27.10 |  | R48A09 | 6 | 0.552 ± 0.186 | 43.30 ± 29.60 |
| R19G03 | 9 | 0.609 ± 0.106 | 33.30 ± 16.10 |  | R48A11 | 9 | 0.565 ± 0.083 | 53.30 ± 23.00 |
| R19G04 | 8 | 0.488 ± 0.083 | 66.70 ± 25.30 |  | R48C06 | 6 | 0.555 ± 0.142 | 50.00 ± 23.40 |
| R19G05 | 6 | 0.521 ± 0.092 | 33.30 ± 18.70 |  | R48C08 | 6 | 0.528 ± 0.116 | 36.70 ± 15.20 |
| **Clumsy cont’** | | | | | | | | |
| **Line name** | **n** | **MaxMeanDist** | **% dead flies** |  | **Line name** | **n** | **MaxMeanDist** | **% dead flies** |
| R19H04 | 6 | 0.496 ± 0.065 | 36.70 ± 16.90 |  | R48E03 | 6 | 0.521 ± 0.114 | 53.30 ± 19.50 |
| R19H09 | 3 | 0.453 ± 0.138 | 33.30 ± 19.20 |  | R48F07 | 3 | 0.466 ± 0.076 | 40.00 ± 23.10 |
| R19H12 | 3 | 0.565 ± 0.044 | 53.30 ± 50.00 |  | R49A10 | 6 | 0.556 ± 0.083 | 68.80 ± 26.30 |
| R20A05 | 3 | 0.695 ± 0.074 | 46.70 ± 22.70 |  | R49C02 | 3 | 0.619 ± 0.056 | 40.00 ± 17.70 |
| R20B07 | 9 | 0.532 ± 0.067 | 50.00 ± 23.10 |  | R49C03 | 3 | 0.408 ± 0.067 | 53.30 ± 12.60 |
| R20D11 | 6 | 0.602 ± 0.032 | 36.70 ± 17.40 |  | R49F03 | 3 | 0.674 ± 0.187 | 46.70 ± 10.20 |
| R20E02 | 5 | 0.664 ± 0.130 | 38.50 ± 18.00 |  | R49H01 | 6 | 0.670 ± 0.098 | 33.30 ± 15.40 |
| R20E06 | 8 | 0.511 ± 0.232 | 40.00 ± 30.90 |  | R51C07 | 17 | 0.697 ± 0.103 | 33.30 ± 26.30 |
| R20F11 | 5 | 0.677 ± 0.036 | 50.00 ± 13.80 |  | R51D12 | 6 | 0.614 ± 0.090 | 56.70 ± 12.40 |
| R20G12 | 12 | 0.403 ± 0.062 | 70.00 ± 22.20 |  | R51E07 | 12 | 0.659 ± 0.168 | 34.10 ± 30.80 |
| R21A04 | 15 | 0.582 ± 0.194 | 33.30 ± 24.90 |  | R51F03 | 3 | 0.506 ± 0.038 | 73.30 ± 23.40 |
| R21B11 | 8 | 0.502 ± 0.053 | 33.90 ± 23.80 |  | R52A06 | 6 | 0.599 ± 0.109 | 89.50 ± 34.90 |
| R21B12 | 11 | 0.682 ± 0.094 | 33.30 ± 17.60 |  | R52B07 | 6 | 0.523 ± 0.128 | 76.70 ± 17.80 |
| R21E05 | 3 | 0.553 ± 0.019 | 33.30 ± 8.50 |  | R52D09 | 3 | 0.653 ± 0.072 | 33.30 ± 16.40 |
| R21H06 | 3 | 0.579 ± 0.093 | 86.70 ± 21.30 |  | R53F03 | 4 | 0.401 ± 0.162 | 35.40 ± 12.80 |
| R21H10 | 9 | 0.674 ± 0.111 | 33.30 ± 23.10 |  | R53G12 | 6 | 0.569 ± 0.078 | 45.20 ± 26.30 |
| R22C08 | 3 | 0.695 ± 0.049 | 40.00 ± 10.20 |  | R54B03 | 6 | 0.657 ± 0.082 | 41.70 ± 15.20 |
| R22E07 | 4 | 0.676 ± 0.096 | 33.30 ± 28.40 |  | R54B06 | 6 | 0.418 ± 0.131 | 44.80 ± 19.10 |
| R22F04 | 6 | 0.667 ± 0.186 | 100.00 ± 5.60 |  | R54E12 | 6 | 0.595 ± 0.077 | 40.00 ± 34.10 |
| R22F12 | 6 | 0.686 ± 0.046 | 63.30 ± 28.70 |  | R55A10 | 3 | 0.668 ± 0.017 | 40.00 ± 10.20 |
| R22G12 | 4 | 0.641 ± 0.120 | 33.30 ± 19.90 |  | R55D02 | 17 | 0.636 ± 0.118 | 93.30 ± 11.80 |
| R23A05 | 10 | 0.661 ± 0.126 | 43.30 ± 33.40 |  | R55F01 | 6 | 0.476 ± 0.075 | 65.50 ± 31.50 |
| R23A07 | 12 | 0.438 ± 0.150 | 33.30 ± 27.40 |  | R55F06 | 7 | 0.559 ± 0.088 | 40.00 ± 27.40 |
| R23B04 | 6 | 0.673 ± 0.172 | 42.90 ± 31.10 |  | R57A07 | 3 | 0.614 ± 0.130 | 33.30 ± 16.80 |
| R23B07 | 3 | 0.486 ± 0.076 | 73.30 ± 40.50 |  | R57E06 | 3 | 0.487 ± 0.118 | 40.00 ± 19.00 |
| R23B09 | 9 | 0.563 ± 0.086 | 60.00 ± 28.00 |  | R57G11 | 9 | 0.665 ± 0.102 | 66.70 ± 16.70 |
| R23D06 | 4 | 0.698 ± 0.059 | 80.00 ± 11.60 |  | R58D04 | 17 | 0.608 ± 0.137 | 57.10 ± 18.30 |
| R23E06 | 3 | 0.593 ± 0.069 | 42.90 ± 39.50 |  | R59C08 | 6 | 0.597 ± 0.087 | 66.70 ± 20.70 |
| R23F04 | 3 | 0.573 ± 0.057 | 40.00 ± 17.70 |  | R59E08 | 5 | 0.537 ± 0.243 | 60.00 ± 14.20 |
| R24D04 | 3 | 0.439 ± 0.056 | 38.50 ± 22.00 |  | R60A10 | 6 | 0.524 ± 0.067 | 100.00 ± 0.00 |
| R24D10 | 10 | 0.431 ± 0.055 | 100.00 ± 0.00 |  | R60G08 | 5 | 0.579 ± 0.143 | 33.30 ± 19.20 |
| R24E10 | 3 | 0.641 ± 0.054 | 46.70 ± 25.70 |  | R61A01 | 3 | 0.636 ± 0.092 | 40.00 ± 20.40 |
| R24F03 | 9 | 0.605 ± 0.065 | 40.00 ± 25.60 |  | R61D08 | 17 | 0.681 ± 0.134 | 86.70 ± 16.40 |
| R24F05 | 3 | 0.549 ± 0.022 | 46.70 ± 13.90 |  | R62A02 | 6 | 0.469 ± 0.137 | 36.70 ± 20.00 |
| R24F09 | 3 | 0.656 ± 0.030 | 33.30 ± 12.90 |  | R64A09 | 6 | 0.456 ± 0.077 | 60.00 ± 21.40 |
| R24G05 | 3 | 0.676 ± 0.055 | 35.70 ± 20.70 |  | R64F02 | 3 | 0.423 ± 0.063 | 46.70 ± 17.60 |
| R24G09 | 6 | 0.581 ± 0.127 | 56.70 ± 28.80 |  | R64G09 | 6 | 0.690 ± 0.096 | 50.00 ± 25.60 |
| R24H05 | 3 | 0.649 ± 0.144 | 53.30 ± 27.80 |  | R65B07 | 18 | 0.536 ± 0.132 | 70.00 ± 16.90 |
| R25B02 | 9 | 0.604 ± 0.136 | 37.50 ± 15.70 |  | R65G02 | 3 | 0.566 ± 0.037 | 35.70 ± 20.20 |
| R25B05 | 6 | 0.443 ± 0.073 | 50.00 ± 33.00 |  | R66B09 | 3 | 0.570 ± 0.093 | 40.00 ± 7.70 |
| R25B08 | 3 | 0.581 ± 0.095 | 33.30 ± 15.40 |  | R67C07 | 22 | 0.539 ± 0.110 | 36.70 ± 19.70 |
| R25C01 | 6 | 0.628 ± 0.075 | 36.70 ± 17.70 |  | R67C09 | 3 | 0.536 ± 0.071 | 46.70 ± 23.40 |
| R25D07 | 3 | 0.676 ± 0.054 | 33.30 ± 22.50 |  | R67D11 | 3 | 0.622 ± 0.052 | 64.30 ± 30.00 |
| R25F07 | 3 | 0.669 ± 0.129 | 38.50 ± 19.70 |  | R67D12 | 3 | 0.539 ± 0.089 | 33.30 ± 41.60 |
| R25F08 | 8 | 0.677 ± 0.183 | 42.90 ± 26.00 |  | R67E01 | 3 | 0.550 ± 0.032 | 53.30 ± 24.00 |
| R26B04 | 3 | 0.686 ± 0.027 | 53.30 ± 17.60 |  | R68B04 | 14 | 0.689 ± 0.152 | 33.30 ± 19.30 |
| R26B07 | 3 | 0.662 ± 0.060 | 35.70 ± 18.10 |  | R68C09 | 6 | 0.709 ± 0.051 | 43.30 ± 25.10 |
| R27F07 | 8 | 0.702 ± 0.094 | 42.80 ± 19.50 |  | R69F08 | 3 | 0.546 ± 0.076 | 40.00 ± 3.90 |
| R27H04 | 3 | 0.599 ± 0.024 | 33.30 ± 13.30 |  | R70E02 | 3 | 0.579 ± 0.116 | 33.30 ± 12.00 |
| R28D05 | 6 | 0.603 ± 0.048 | 33.30 ± 14.60 |  | R70H06 | 6 | 0.573 ± 0.057 | 33.30 ± 13.40 |
| R29E02 | 9 | 0.608 ± 0.168 | 60.00 ± 31.00 |  | R71B08 | 6 | 0.551 ± 0.119 | 46.70 ± 20.00 |
| R29E10 | 15 | 0.650 ± 0.102 | 38.50 ± 21.60 |  | R71C06 | 6 | 0.616 ± 0.099 | 48.60 ± 23.30 |
| R30A08 | 3 | 0.671 ± 0.073 | 40.00 ± 23.10 |  | R71G04 | 6 | 0.529 ± 0.084 | 46.70 ± 16.80 |
| R30A09 | 6 | 0.624 ± 0.066 | 76.70 ± 19.20 |  | R72A03 | 6 | 0.679 ± 0.071 | 56.70 ± 18.00 |
| R30B02 | 6 | 0.555 ± 0.130 | 43.30 ± 23.80 |  | R72B07 | 9 | 0.609 ± 0.182 | 33.30 ± 14.60 |
| R30B05 | 6 | 0.537 ± 0.072 | 36.70 ± 25.90 |  | R72H11 | 9 | 0.601 ± 0.093 | 71.40 ± 20.70 |
| R30F09 | 6 | 0.699 ± 0.149 | 100.00 ± 0.00 |  | R74F07 | 13 | 0.482 ± 0.161 | 33.30 ± 23.80 |
| R31A04 | 3 | 0.683 ± 0.036 | 66.70 ± 6.70 |  | R75B02 | 6 | 0.526 ± 0.078 | 33.30 ± 13.10 |
| R31A10 | 6 | 0.483 ± 0.053 | 40.00 ± 22.20 |  | R76F01 | 6 | 0.551 ± 0.139 | 33.30 ± 17.10 |
| R31B12 | 5 | 0.647 ± 0.179 | 100.00 ± 43.60 |  | R77D01 | 9 | 0.533 ± 0.139 | 40.00 ± 22.70 |
| **Clumsy cont’** | | | | | | | | |
| **Line name** | **n** | **MaxMeanDist** | **% dead flies** |  | **Line name** | **n** | **MaxMeanDist** | **% dead flies** |
| R32A08 | 9 | 0.510 ± 0.178 | 38.50 ± 23.00 |  | R77E01 | 6 | 0.647 ± 0.059 | 43.30 ± 17.20 |
| R32D08 | 3 | 0.676 ± 0.099 | 60.00 ± 6.70 |  | R78B07 | 12 | 0.679 ± 0.128 | 79.30 ± 16.70 |
| R32E05 | 3 | 0.657 ± 0.088 | 33.30 ± 24.00 |  | R78F01 | 6 | 0.636 ± 0.085 | 33.30 ± 22.90 |
| R33A01 | 3 | 0.575 ± 0.199 | 38.50 ± 10.10 |  | R79C09 | 3 | 0.414 ± 0.095 | 53.30 ± 20.60 |
| R33D07 | 6 | 0.604 ± 0.108 | 60.00 ± 25.70 |  | R79E02 | 9 | 0.686 ± 0.130 | 73.30 ± 20.40 |
| R33E05 | 3 | 0.562 ± 0.072 | 60.00 ± 35.20 |  | R79G10 | 6 | 0.604 ± 0.086 | 40.00 ± 24.00 |
| R34C04 | 6 | 0.587 ± 0.111 | 38.30 ± 24.70 |  | R80B04 | 6 | 0.686 ± 0.117 | 43.30 ± 20.50 |
| R34D03 | 6 | 0.606 ± 0.098 | 60.00 ± 29.60 |  | R80C06 | 6 | 0.626 ± 0.047 | 35.70 ± 23.00 |
| R34E01 | 9 | 0.563 ± 0.088 | 40.00 ± 21.60 |  | R80G09 | 3 | 0.587 ± 0.080 | 40.00 ± 26.70 |
| R34G02 | 6 | 0.574 ± 0.084 | 33.30 ± 26.70 |  | R82F10 | 17 | 0.544 ± 0.108 | 33.30 ± 23.60 |
| R34G05 | 6 | 0.548 ± 0.050 | 72.40 ± 32.20 |  | R85A07 | 6 | 0.588 ± 0.138 | 63.30 ± 41.00 |
| R35C07 | 8 | 0.571 ± 0.096 | 86.20 ± 37.90 |  | R85C10 | 6 | 0.651 ± 0.042 | 76.70 ± 42.90 |
| R35D03 | 3 | 0.709 ± 0.204 | 33.30 ± 18.80 |  | R85F01 | 3 | 0.517 ± 0.145 | 46.70 ± 27.00 |
| R35F12 | 3 | 0.397 ± 0.051 | 100.00 ± 3.90 |  | R86D03 | 6 | 0.631 ± 0.161 | 40.00 ± 24.40 |
| R36G02 | 9 | 0.620 ± 0.144 | 60.00 ± 31.70 |  | R86D05 | 12 | 0.659 ± 0.176 | 36.70 ± 24.20 |
| R37A12 | 6 | 0.595 ± 0.091 | 56.70 ± 33.30 |  | R87F10 | 3 | 0.629 ± 0.036 | 60.00 ± 27.70 |
| R37G03 | 5 | 0.638 ± 0.125 | 80.00 ± 22.50 |  | R89A08 | 8 | 0.491 ± 0.120 | 36.70 ± 21.70 |
| R38A08 | 6 | 0.622 ± 0.123 | 41.20 ± 29.30 |  | R89D06 | 9 | 0.564 ± 0.088 | 40.00 ± 17.80 |
| R38B04 | 6 | 0.408 ± 0.111 | 40.00 ± 30.00 |  | R89E07 | 6 | 0.650 ± 0.131 | 37.90 ± 33.20 |
| R38G10 | 6 | 0.608 ± 0.073 | 40.00 ± 18.90 |  | R89E09 | 6 | 0.497 ± 0.195 | 36.70 ± 39.40 |
| R39B11 | 5 | 0.529 ± 0.085 | 33.30 ± 19.60 |  | R91B03 | 6 | 0.575 ± 0.091 | 63.30 ± 40.20 |
| R39D07 | 3 | 0.556 ± 0.138 | 33.30 ± 34.90 |  | R92B03 | 9 | 0.503 ± 0.052 | 46.70 ± 24.30 |
| R39G09 | 6 | 0.441 ± 0.045 | 43.30 ± 34.70 |  | R92C03 | 6 | 0.567 ± 0.066 | 33.30 ± 20.90 |
| R39H07 | 6 | 0.601 ± 0.103 | 46.70 ± 27.20 |  | R92D04 | 6 | 0.527 ± 0.134 | 53.30 ± 27.50 |
| R39H08 | 9 | 0.589 ± 0.118 | 33.30 ± 31.90 |  | R92G07 | 6 | 0.535 ± 0.143 | 33.30 ± 36.10 |
| R40C01 | 9 | 0.559 ± 0.095 | 53.30 ± 23.40 |  | R94A07 | 6 | 0.502 ± 0.070 | 36.70 ± 19.10 |
| R40C08 | 6 | 0.533 ± 0.088 | 36.70 ± 16.80 |  | R94B04 | 5 | 0.604 ± 0.058 | 33.30 ± 33.10 |
| R40D04 | 3 | 0.647 ± 0.101 | 35.70 ± 14.30 |  | R94C05 | 9 | 0.557 ± 0.110 | 33.30 ± 14.90 |
| R40E02 | 3 | 0.693 ± 0.078 | 53.30 ± 19.00 |  | R95E11 | 3 | 0.587 ± 0.025 | 33.30 ± 27.30 |
| R40E10 | 3 | 0.697 ± 0.090 | 50.00 ± 17.10 |  | R95H02 | 6 | 0.582 ± 0.077 | 46.70 ± 27.80 |
| R40G09 | 6 | 0.610 ± 0.090 | 60.00 ± 19.80 |  |  |  |  |  |
| **Overeager** | | | | | | | | |
| **Line name** | **n** | **MaxMeanDist** | **% dead flies** |  | **Line name** | **n** | **MaxMeanDist** | **% dead flies** |
| R10A11 | 3 | 0.716 ± 0.027 | 40.00 ± 17.70 |  | R27H08 | 6 | 0.780 ± 0.125 | 36.67 ± 14.40 |
| R12C07 | 6 | 0.717 ± 0.153 | 83.33 ± 44.10 |  | R30C02 | 3 | 0.804 ± 0.117 | 73.33 ± 21.40 |
| R16A05 | 3 | 0.730 ± 0.030 | 40.00 ± 21.80 |  | R30C08 | 3 | 0.732 ± 0.054 | 66.67 ± 21.40 |
| R20B02 | 4 | 0.806 ± 0.088 | 35.83 ± 24.10 |  | R30H05 | 3 | 0.773 ± 0.032 | 53.85 ± 21.30 |
| R20E03 | 15 | 0.737 ± 0.096 | 33.33 ± 22.30 |  | R38A03 | 3 | 0.724 ± 0.063 | 40.00 ± 6.70 |
| R21D06 | 5 | 0.793 ± 0.077 | 46.67 ± 29.60 |  | R38H01 | 6 | 0.715 ± 0.070 | 41.67 ± 20.50 |
| R21H09 | 10 | 0.770 ± 0.094 | 63.33 ± 15.50 |  | R49F09 | 17 | 0.762 ± 0.111 | 33.33 ± 20.10 |
| R22A04 | 3 | 0.804 ± 0.108 | 43.75 ± 16.70 |  | R52D11 | 6 | 0.739 ± 0.105 | 36.67 ± 11.20 |
| R22F08 | 4 | 0.739 ± 0.000 | 33.33 ± 22.30 |  | R55B01 | 16 | 0.762 ± 0.187 | 48.33 ± 24.90 |
| R22G03 | 4 | 0.753 ± 0.057 | 46.67 ± 17.60 |  | R65A10 | 12 | 0.775 ± 0.106 | 33.33 ± 20.90 |
| R22H11 | 4 | 0.791 ± 0.056 | 56.67 ± 16.40 |  | R65B08 | 15 | 0.741 ± 0.120 | 40.00 ± 25.00 |
| R23A11 | 3 | 0.749 ± 0.033 | 33.33 ± 13.90 |  | R69C01 | 18 | 0.772 ± 0.136 | 46.67 ± 23.20 |
| R23A12 | 3 | 0.800 ± 0.049 | 33.33 ± 28.70 |  | R69H11 | 18 | 0.754 ± 0.099 | 40.00 ± 24.10 |
| R23D10 | 4 | 0.778 ± 0.042 | 36.67 ± 27.40 |  | R77F03 | 5 | 0.821 ± 0.114 | 40.00 ± 25.80 |
| R23E02 | 3 | 0.837 ± 0.102 | 40.00 ± 5.80 |  | R79F06 | 12 | 0.787 ± 0.139 | 45.90 ± 25.80 |
| R25D03 | 3 | 0.765 ± 0.135 | 42.86 ± 23.00 |  | R87D07 | 13 | 0.724 ± 0.134 | 60.00 ± 27.10 |
| R25D08 | 6 | 0.763 ± 0.044 | 36.67 ± 17.10 |  | R89H03 | 6 | 0.763 ± 0.126 | 36.67 ± 14.40 |
| R26F03 | 10 | 0.730 ± 0.079 | 41.43 ± 23.40 |  |  |  |  |  |
| **Super Climber** | | | | | | | | |
| **Line name** | **n** | **MaxMeanDist** | **% dead flies** |  | **Line name** | **n** | **MaxMeanDist** | **% dead flies** |
| R09C12 | 6 | 0.710 ± 0.139 | 13.33 ± 25.20 |  | R64C05 | 12 | 0.760 ± 0.111 | 20.00 ± 18.00 |
| R10B02 | 8 | 0.728 ± 0.145 | 13.33 ± 4.70 |  | R64C10 | 10 | 0.732 ± 0.121 | 10.00 ± 21.60 |
| R10B10 | 5 | 0.735 ± 0.147 | 13.33 ± 7.60 |  | R64C11 | 17 | 0.758 ± 0.181 | 27.27 ± 20.60 |
| R12C01 | 7 | 0.716 ± 0.042 | 26.67 ± 25.30 |  | R64C12 | 15 | 0.775 ± 0.143 | 26.67 ± 16.90 |
| R14C07 | 3 | 0.773 ± 0.245 | 13.33 ± 10.20 |  | R64E01 | 12 | 0.711 ± 0.142 | 26.67 ± 32.40 |
| R14D12 | 3 | 0.726 ± 0.077 | 14.29 ± 11.30 |  | R64E12 | 17 | 0.710 ± 0.159 | 7.14 ± 23.40 |
| **Super Climber cont’** | | | | | | | | |
| **Line name** | **n** | **MaxMeanDist** | **% dead flies** |  | **Line name** | **n** | **MaxMeanDist** | **% dead flies** |
| R16E05 | 3 | 0.755 ± 0.048 | 6.67 ± 4.40 |  | R64F06 | 12 | 0.814 ± 0.084 | 20.00 ± 26.00 |
| R17C11 | 3 | 0.728 ± 0.203 | 13.33 ± 6.70 |  | R64F08 | 12 | 0.733 ± 0.103 | 26.67 ± 17.60 |
| R18B02 | 3 | 0.713 ± 0.179 | 0.00 ± 0.00 |  | R64G01 | 18 | 0.715 ± 0.157 | 20.00 ± 18.40 |
| R18B08 | 3 | 0.732 ± 0.042 | 0.00 ± 30.80 |  | R65C03 | 15 | 0.738 ± 0.093 | 13.33 ± 9.50 |
| R18C04 | 3 | 0.715 ± 0.019 | 0.00 ± 27.00 |  | R65E10 | 12 | 0.725 ± 0.159 | 13.33 ± 23.30 |
| R19G02 | 3 | 0.723 ± 0.328 | 0.00 ± 7.70 |  | R65E11 | 7 | 0.759 ± 0.145 | 0.00 ± 6.40 |
| R19G12 | 3 | 0.734 ± 0.164 | 6.67 ± 6.70 |  | R65G07 | 15 | 0.728 ± 0.161 | 20.00 ± 21.10 |
| R20A07 | 6 | 0.725 ± 0.047 | 16.67 ± 20.40 |  | R67A11 | 12 | 0.714 ± 0.150 | 13.33 ± 10.80 |
| R20A10 | 3 | 0.784 ± 0.082 | 0.00 ± 11.50 |  | R67C08 | 10 | 0.757 ± 0.097 | 10.00 ± 10.50 |
| R20E08 | 7 | 0.717 ± 0.091 | 13.33 ± 14.90 |  | R67D09 | 6 | 0.715 ± 0.101 | 23.33 ± 22.20 |
| R20G03 | 5 | 0.819 ± 0.156 | 26.67 ± 27.50 |  | R67F03 | 13 | 0.795 ± 0.189 | 13.33 ± 11.50 |
| R21A03 | 3 | 0.767 ± 0.093 | 25.00 ± 26.80 |  | R68A08 | 14 | 0.740 ± 0.197 | 8.71 ± 16.90 |
| R21D03 | 10 | 0.720 ± 0.133 | 23.33 ± 13.70 |  | R68A11 | 18 | 0.748 ± 0.166 | 17.14 ± 18.80 |
| R21F10 | 4 | 0.724 ± 0.044 | 10.00 ± 20.70 |  | R68B03 | 11 | 0.797 ± 0.058 | 6.67 ± 22.50 |
| R21H12 | 9 | 0.719 ± 0.139 | 13.33 ± 25.00 |  | R68C02 | 17 | 0.785 ± 0.180 | 6.67 ± 18.80 |
| R22A06 | 3 | 0.757 ± 0.054 | 6.67 ± 3.90 |  | R68C06 | 3 | 0.743 ± 0.092 | 13.33 ± 7.70 |
| R22A09 | 6 | 0.761 ± 0.077 | 13.33 ± 14.40 |  | R68C07 | 9 | 0.724 ± 0.127 | 13.33 ± 22.10 |
| R22A11 | 4 | 0.763 ± 0.030 | 13.33 ± 3.50 |  | R69A12 | 9 | 0.770 ± 0.064 | 13.33 ± 16.30 |
| R22A12 | 4 | 0.795 ± 0.063 | 10.51 ± 6.70 |  | R69C04 | 12 | 0.794 ± 0.053 | 6.90 ± 31.00 |
| R22B08 | 3 | 0.820 ± 0.097 | 7.14 ± 4.10 |  | R69F02 | 12 | 0.721 ± 0.143 | 13.33 ± 9.50 |
| R22E04 | 11 | 0.757 ± 0.101 | 21.43 ± 19.50 |  | R69F07 | 16 | 0.763 ± 0.181 | 2.94 ± 23.80 |
| R22F05 | 13 | 0.733 ± 0.238 | 26.67 ± 30.80 |  | R69F11 | 7 | 0.763 ± 0.058 | 6.67 ± 18.80 |
| R22H02 | 7 | 0.755 ± 0.152 | 13.33 ± 7.70 |  | R70B04 | 17 | 0.781 ± 0.138 | 13.33 ± 20.10 |
| R22H05 | 3 | 0.755 ± 0.131 | 14.29 ± 25.00 |  | R70B11 | 12 | 0.736 ± 0.095 | 9.58 ± 10.70 |
| R22H06 | 4 | 0.782 ± 0.068 | 13.33 ± 8.40 |  | R70B12 | 11 | 0.723 ± 0.087 | 13.33 ± 15.70 |
| R23B06 | 4 | 0.787 ± 0.101 | 13.33 ± 32.30 |  | R70C03 | 9 | 0.734 ± 0.097 | 13.33 ± 21.60 |
| R23B10 | 4 | 0.806 ± 0.120 | 13.57 ± 18.70 |  | R70E08 | 14 | 0.735 ± 0.156 | 13.33 ± 18.30 |
| R23C01 | 4 | 0.784 ± 0.090 | 3.33 ± 4.20 |  | R70F10 | 6 | 0.741 ± 0.190 | 23.33 ± 17.20 |
| R23C03 | 7 | 0.802 ± 0.249 | 6.67 ± 21.00 |  | R70G08 | 11 | 0.712 ± 0.072 | 6.67 ± 14.80 |
| R23C07 | 8 | 0.715 ± 0.113 | 13.33 ± 14.20 |  | R70H05 | 6 | 0.730 ± 0.168 | 30.00 ± 11.70 |
| R23C12 | 6 | 0.816 ± 0.099 | 17.86 ± 22.60 |  | R71D05 | 12 | 0.766 ± 0.136 | 6.67 ± 16.00 |
| R23D02 | 7 | 0.735 ± 0.117 | 20.00 ± 10.50 |  | R71E06 | 14 | 0.795 ± 0.167 | 0.00 ± 20.30 |
| R23D04 | 4 | 0.780 ± 0.096 | 10.83 ± 6.30 |  | R71F10 | 15 | 0.760 ± 0.137 | 13.33 ± 15.60 |
| R23D05 | 4 | 0.774 ± 0.092 | 14.36 ± 8.30 |  | R72C11 | 13 | 0.766 ± 0.109 | 14.29 ± 30.00 |
| R23D07 | 4 | 0.805 ± 0.048 | 0.00 ± 13.40 |  | R72D01 | 14 | 0.739 ± 0.113 | 13.33 ± 22.90 |
| R23D08 | 4 | 0.825 ± 0.037 | 30.00 ± 11.70 |  | R72E01 | 6 | 0.751 ± 0.177 | 27.62 ± 16.80 |
| R23D09 | 4 | 0.786 ± 0.047 | 22.25 ± 12.10 |  | R72E05 | 18 | 0.798 ± 0.107 | 13.33 ± 18.20 |
| R23D11 | 3 | 0.770 ± 0.086 | 21.43 ± 16.70 |  | R72E10 | 12 | 0.761 ± 0.113 | 6.67 ± 23.20 |
| R23E01 | 3 | 0.718 ± 0.051 | 23.08 ± 6.90 |  | R72G03 | 9 | 0.786 ± 0.080 | 13.33 ± 14.20 |
| R23E03 | 3 | 0.773 ± 0.055 | 28.57 ± 25.10 |  | R72G07 | 14 | 0.759 ± 0.172 | 10.24 ± 10.10 |
| R24C10 | 3 | 0.857 ± 0.046 | 6.67 ± 19.20 |  | R72G12 | 15 | 0.729 ± 0.108 | 13.33 ± 11.30 |
| R24D03 | 3 | 0.797 ± 0.030 | 0.00 ± 11.50 |  | R72H06 | 14 | 0.805 ± 0.107 | 30.00 ± 24.10 |
| R24D09 | 3 | 0.776 ± 0.055 | 0.00 ± 3.90 |  | R73A05 | 18 | 0.761 ± 0.147 | 10.00 ± 11.00 |
| R24E01 | 3 | 0.764 ± 0.054 | 0.00 ± 3.90 |  | R73B05 | 9 | 0.721 ± 0.082 | 6.67 ± 15.60 |
| R25A04 | 3 | 0.720 ± 0.210 | 26.67 ± 16.00 |  | R73C04 | 3 | 0.736 ± 0.027 | 6.67 ± 7.70 |
| R25B04 | 3 | 0.736 ± 0.016 | 21.43 ± 10.10 |  | R73E01 | 12 | 0.778 ± 0.075 | 10.24 ± 19.10 |
| R25D06 | 3 | 0.788 ± 0.144 | 20.00 ± 13.30 |  | R73F07 | 14 | 0.785 ± 0.123 | 20.00 ± 16.70 |
| R25D11 | 3 | 0.801 ± 0.058 | 7.14 ± 30.70 |  | R73H09 | 9 | 0.745 ± 0.086 | 13.33 ± 13.40 |
| R25H10 | 3 | 0.753 ± 0.060 | 13.33 ± 15.40 |  | R74B06 | 12 | 0.718 ± 0.135 | 16.67 ± 13.90 |
| R26A02 | 6 | 0.710 ± 0.152 | 32.56 ± 17.40 |  | R74G01 | 15 | 0.797 ± 0.167 | 26.67 ± 27.50 |
| R26E09 | 3 | 0.729 ± 0.092 | 26.67 ± 34.50 |  | R75A11 | 9 | 0.786 ± 0.138 | 6.67 ± 5.20 |
| R27A07 | 3 | 0.728 ± 0.103 | 0.00 ± 3.90 |  | R75C10 | 8 | 0.711 ± 0.061 | 0.00 ± 7.80 |
| R27G01 | 3 | 0.763 ± 0.089 | 6.67 ± 6.70 |  | R75F02 | 17 | 0.763 ± 0.157 | 13.33 ± 12.20 |
| R28A09 | 3 | 0.744 ± 0.128 | 0.00 ± 4.10 |  | R75F05 | 11 | 0.772 ± 0.163 | 0.00 ± 4.60 |
| R28C05 | 6 | 0.735 ± 0.207 | 16.67 ± 7.00 |  | R75G12 | 3 | 0.799 ± 0.173 | 13.33 ± 20.40 |
| R30E10 | 6 | 0.716 ± 0.160 | 26.67 ± 15.60 |  | R76C01 | 12 | 0.722 ± 0.134 | 0.00 ± 9.00 |
| R30F11 | 3 | 0.716 ± 0.093 | 13.33 ± 23.30 |  | R76D11 | 13 | 0.752 ± 0.109 | 13.33 ± 19.60 |
| R31A09 | 6 | 0.717 ± 0.069 | 16.67 ± 18.10 |  | R77A06 | 15 | 0.802 ± 0.132 | 6.67 ± 6.90 |
| R40D05 | 3 | 0.774 ± 0.186 | 13.33 ± 30.80 |  | R78A01 | 16 | 0.759 ± 0.190 | 8.12 ± 10.70 |
| R43F05 | 3 | 0.774 ± 0.119 | 6.67 ± 6.70 |  | R78D12 | 12 | 0.735 ± 0.131 | 13.33 ± 13.20 |
| **Super Climber cont’** | | | | | | | | |
| **Line name** | **n** | **MaxMeanDist** | **% dead flies** |  | **Line name** | **n** | **MaxMeanDist** | **% dead flies** |
| R45E04 | 6 | 0.710 ± 0.111 | 3.12 ± 8.30 |  | R78E02 | 9 | 0.819 ± 0.118 | 6.67 ± 10.40 |
| R46F08 | 14 | 0.738 ± 0.173 | 20.00 ± 22.40 |  | R78H01 | 9 | 0.727 ± 0.137 | 20.00 ± 20.50 |
| R47B12 | 3 | 0.745 ± 0.098 | 14.29 ± 20.30 |  | R79B09 | 11 | 0.712 ± 0.088 | 6.67 ± 6.70 |
| R49C04 | 6 | 0.716 ± 0.105 | 30.95 ± 16.60 |  | R79D09 | 15 | 0.746 ± 0.180 | 21.43 ± 13.90 |
| R50B07 | 11 | 0.753 ± 0.080 | 6.67 ± 22.50 |  | R79F07 | 8 | 0.742 ± 0.202 | 6.67 ± 5.40 |
| R50F12 | 14 | 0.715 ± 0.153 | 6.90 ± 17.00 |  | R80A01 | 18 | 0.729 ± 0.198 | 13.33 ± 9.10 |
| R50G08 | 3 | 0.728 ± 0.131 | 26.67 ± 13.90 |  | R80E06 | 6 | 0.758 ± 0.103 | 27.62 ± 19.30 |
| R51C06 | 12 | 0.716 ± 0.112 | 26.67 ± 24.20 |  | R80H02 | 3 | 0.817 ± 0.221 | 6.67 ± 26.90 |
| R51D11 | 10 | 0.763 ± 0.086 | 13.33 ± 23.00 |  | R82A03 | 15 | 0.728 ± 0.184 | 6.67 ± 11.00 |
| R51G03 | 15 | 0.751 ± 0.145 | 0.00 ± 9.80 |  | R82B09 | 18 | 0.754 ± 0.154 | 7.88 ± 9.20 |
| R51H09 | 15 | 0.720 ± 0.104 | 13.33 ± 10.20 |  | R82C09 | 11 | 0.781 ± 0.101 | 26.67 ± 26.00 |
| R52F05 | 11 | 0.738 ± 0.073 | 20.00 ± 28.30 |  | R82E12 | 14 | 0.759 ± 0.122 | 6.67 ± 7.40 |
| R52F10 | 12 | 0.729 ± 0.067 | 9.58 ± 10.40 |  | R82F11 | 18 | 0.737 ± 0.150 | 16.67 ± 13.80 |
| R52H03 | 17 | 0.742 ± 0.130 | 13.33 ± 22.00 |  | R82G02 | 15 | 0.731 ± 0.167 | 6.67 ± 18.40 |
| R53C07 | 15 | 0.741 ± 0.188 | 0.00 ± 6.60 |  | R83C03 | 14 | 0.711 ± 0.160 | 17.14 ± 17.00 |
| R54C01 | 11 | 0.759 ± 0.113 | 0.00 ± 8.10 |  | R83H01 | 13 | 0.736 ± 0.181 | 6.67 ± 17.20 |
| R54D12 | 3 | 0.809 ± 0.059 | 26.67 ± 27.80 |  | R83H05 | 11 | 0.725 ± 0.146 | 13.33 ± 10.50 |
| R54F05 | 15 | 0.753 ± 0.096 | 6.67 ± 14.00 |  | R84B09 | 15 | 0.739 ± 0.170 | 6.67 ± 13.00 |
| R54G03 | 11 | 0.723 ± 0.088 | 7.14 ± 14.90 |  | R84B11 | 8 | 0.731 ± 0.140 | 0.00 ± 9.60 |
| R54G12 | 19 | 0.758 ± 0.205 | 20.00 ± 22.80 |  | R84C02 | 17 | 0.721 ± 0.258 | 6.67 ± 11.60 |
| R54H01 | 14 | 0.755 ± 0.120 | 30.00 ± 16.60 |  | R84H08 | 12 | 0.781 ± 0.119 | 10.24 ± 24.80 |
| R54H08 | 12 | 0.777 ± 0.129 | 6.67 ± 6.40 |  | R85B06 | 11 | 0.745 ± 0.102 | 13.33 ± 22.90 |
| R54H09 | 12 | 0.721 ± 0.101 | 10.00 ± 12.40 |  | R85D07 | 5 | 0.747 ± 0.153 | 13.33 ± 20.70 |
| R54H11 | 17 | 0.746 ± 0.136 | 21.43 ± 23.50 |  | R85F11 | 14 | 0.735 ± 0.140 | 13.33 ± 16.20 |
| R55D11 | 14 | 0.726 ± 0.130 | 10.00 ± 15.60 |  | R86G01 | 8 | 0.732 ± 0.141 | 10.00 ± 16.80 |
| R55F08 | 14 | 0.752 ± 0.081 | 6.67 ± 12.20 |  | R86G03 | 17 | 0.735 ± 0.111 | 6.67 ± 8.70 |
| R55H04 | 11 | 0.804 ± 0.169 | 20.00 ± 19.80 |  | R86H04 | 13 | 0.733 ± 0.153 | 12.50 ± 22.10 |
| R56B05 | 15 | 0.718 ± 0.103 | 13.33 ± 13.10 |  | R86H12 | 4 | 0.756 ± 0.068 | 6.67 ± 3.30 |
| R56C08 | 16 | 0.715 ± 0.158 | 6.67 ± 16.80 |  | R87B02 | 11 | 0.747 ± 0.097 | 20.00 ± 17.70 |
| R56D02 | 12 | 0.726 ± 0.075 | 10.00 ± 13.70 |  | R87B03 | 10 | 0.799 ± 0.223 | 16.67 ± 18.80 |
| R56E10 | 15 | 0.717 ± 0.241 | 20.00 ± 18.40 |  | R87E02 | 10 | 0.732 ± 0.127 | 0.00 ± 18.90 |
| R57A12 | 7 | 0.734 ± 0.095 | 6.67 ± 9.20 |  | R87F12 | 3 | 0.811 ± 0.062 | 13.33 ± 3.80 |
| R57B02 | 12 | 0.758 ± 0.157 | 6.67 ± 9.80 |  | R89A03 | 3 | 0.854 ± 0.081 | 13.33 ± 11.50 |
| R57H02 | 12 | 0.767 ± 0.085 | 0.00 ± 4.50 |  | R89G04 | 3 | 0.743 ± 0.091 | 6.67 ± 6.70 |
| R58G03 | 15 | 0.714 ± 0.160 | 8.33 ± 14.70 |  | R91A01 | 3 | 0.735 ± 0.284 | 6.67 ± 6.70 |
| R60B12 | 11 | 0.710 ± 0.151 | 14.29 ± 10.90 |  | R92C04 | 3 | 0.853 ± 0.033 | 20.00 ± 10.20 |
| R60E12 | 14 | 0.716 ± 0.130 | 10.24 ± 11.40 |  | R94B10 | 3 | 0.738 ± 0.391 | 0.00 ± 3.90 |
| R61E02 | 11 | 0.725 ± 0.103 | 26.67 ± 26.00 |  |  |  |  |  |

**Supplementary Table 2** **List of lines with altered time taken to reach the maximum mean distance from centre in the ring-gap experiments of Figure 1 as shown in the evaluation of Supplementary Fig. S1a .** Lines that took less than 500 s are listed here.

| **Line name** | **n** | **TimeToMax[s]** |  | **Line name** | **n** | **TimeToMax[s]** |  | **Line name** | **n** | **TimeToMax[s]** |
| --- | --- | --- | --- | --- | --- | --- | --- | --- | --- | --- |
| R09A11 | 3 | 480.0 ± 56.8 |  | R21H02 | 3 | 390.0 ± 158.0 |  | R33C06 | 6 | 465.0 ± 210.4 |
| R10B03 | 3 | 375.0 ± 98.4 |  | R22A02 | 14 | 480.0 ± 157.9 |  | R34B03 | 3 | 465.0 ± 112.6 |
| R10B10 | 5 | 450.0 ± 79.7 |  | R22A05 | 3 | 450.0 ± 74.0 |  | R34D03 | 6 | 480.0 ± 102.4 |
| R10E07 | 12 | 465.0 ± 143.5 |  | R22E06 | 7 | 465.0 ± 112.9 |  | R34E05 | 3 | 465.0 ± 56.8 |
| R10G01 | 6 | 480.0 ± 51.1 |  | R22F04 | 6 | 420.0 ± 188.7 |  | R34E11 | 3 | 360.0 ± 144.1 |
| R10G03 | 3 | 450.0 ± 31.2 |  | R22F06 | 3 | 435.0 ± 83.5 |  | R34G05 | 6 | 450.0 ± 79.0 |
| R10H09 | 3 | 405.0 ± 119.1 |  | R22H04 | 4 | 442.5 ± 78.4 |  | R35C07 | 8 | 472.5 ± 133.2 |
| R11B06 | 6 | 472.5 ± 103.1 |  | R22H05 | 3 | 465.0 ± 199.7 |  | R35F12 | 3 | 300.0 ± 165.2 |
| R11D01 | 3 | 480.0 ± 95.3 |  | R23A12 | 3 | 480.0 ± 30.0 |  | R37G03 | 5 | 405.0 ± 101.0 |
| R11E12 | 3 | 180.0 ± 34.6 |  | R23B07 | 3 | 465.0 ± 167.0 |  | R38A04 | 6 | 457.5 ± 166.1 |
| R11F06 | 3 | 480.0 ± 39.7 |  | R23B11 | 3 | 480.0 ± 70.9 |  | R38B09 | 3 | 465.0 ± 74.0 |
| R11G06 | 6 | 480.0 ± 108.0 |  | R23D02 | 7 | 450.0 ± 104.2 |  | R38G04 | 3 | 450.0 ± 39.7 |
| R12B09 | 13 | 480.0 ± 72.5 |  | R23D10 | 4 | 487.5 ± 59.2 |  | R39H10 | 3 | 435.0 ± 82.6 |
| R12C11 | 5 | 465.0 ± 62.2 |  | R23E03 | 3 | 450.0 ± 93.7 |  | R40E10 | 3 | 465.0 ± 65.4 |
| R13C04 | 9 | 480.0 ± 66.7 |  | R23E04 | 6 | 472.5 ± 49.7 |  | R42F03 | 3 | 150.0 ± 169.0 |
| R13D09 | 3 | 435.0 ± 91.7 |  | R23E08 | 3 | 420.0 ± 93.7 |  | R43F05 | 3 | 435.0 ± 54.1 |
| R14C06 | 3 | 480.0 ± 77.0 |  | R23H09 | 3 | 405.0 ± 131.1 |  | R44C02 | 5 | 465.0 ± 22.2 |
| R14E10 | 3 | 435.0 ± 77.9 |  | R24C05 | 3 | 480.0 ± 70.9 |  | R44E04 | 9 | 450.0 ± 108.7 |
| R14H09 | 3 | 435.0 ± 60.6 |  | R24C12 | 8 | 412.5 ± 122.6 |  | R49B07 | 6 | 487.5 ± 81.5 |
| R15A09 | 3 | 420.0 ± 93.7 |  | R24D03 | 3 | 450.0 ± 120.3 |  | R49C01 | 3 | 480.0 ± 60.6 |
| R15C01 | 3 | 390.0 ± 127.6 |  | R24D05 | 3 | 345.0 ± 294.1 |  | R49C04 | 6 | 480.0 ± 114.8 |
| R15C12 | 6 | 480.0 ± 86.4 |  | R24D09 | 3 | 450.0 ± 98.4 |  | R49F03 | 3 | 435.0 ± 113.2 |
| R16D07 | 3 | 135.0 ± 199.7 |  | R24D10 | 10 | 247.5 ± 217.3 |  | R49G06 | 3 | 480.0 ± 52.7 |
| R16H03 | 5 | 375.0 ± 212.4 |  | R24F12 | 3 | 465.0 ± 85.3 |  | R49G08 | 6 | 487.5 ± 110.1 |
| R17C08 | 3 | 405.0 ± 131.1 |  | R24G05 | 3 | 465.0 ± 65.4 |  | R50F04 | 12 | 487.5 ± 93.2 |
| R17D03 | 4 | 367.5 ± 248.3 |  | R24G10 | 3 | 480.0 ± 68.7 |  | R50G03 | 15 | 480.0 ± 66.6 |
| R17E03 | 3 | 420.0 ± 70.9 |  | R24H02 | 6 | 435.0 ± 87.3 |  | R51C01 | 6 | 240.0 ± 218.0 |
| R17G02 | 3 | 465.0 ± 79.4 |  | R24H04 | 3 | 390.0 ± 91.2 |  | R51C07 | 17 | 465.0 ± 119.1 |
| R17H10 | 8 | 435.0 ± 176.2 |  | R24H08 | 3 | 390.0 ± 112.6 |  | R51G10 | 3 | 480.0 ± 90.4 |
| R18C02 | 3 | 480.0 ± 90.4 |  | R25A05 | 3 | 465.0 ± 98.4 |  | R51H05 | 18 | 480.0 ± 117.0 |
| R18D02 | 3 | 420.0 ± 135.3 |  | R25B02 | 9 | 480.0 ± 55.6 |  | R52A06 | 6 | 450.0 ± 80.9 |
| R18D04 | 9 | 435.0 ± 87.1 |  | R25B11 | 3 | 480.0 ± 68.7 |  | R52F01 | 3 | 480.0 ± 54.1 |
| R18D06 | 3 | 465.0 ± 68.7 |  | R25D07 | 3 | 450.0 ± 45.8 |  | R52F05 | 11 | 480.0 ± 103.8 |
| R18G02 | 3 | 465.0 ± 167.0 |  | R25D10 | 9 | 480.0 ± 66.6 |  | R52H01 | 3 | 255.0 ± 124.9 |
| R19B03 | 6 | 442.5 ± 110.4 |  | R25D12 | 3 | 465.0 ± 98.4 |  | R54A12 | 3 | 480.0 ± 65.4 |
| R19G04 | 8 | 487.5 ± 159.9 |  | R25F07 | 3 | 480.0 ± 48.2 |  | R54C01 | 11 | 465.0 ± 97.2 |
| R19G12 | 3 | 465.0 ± 77.0 |  | R25H10 | 3 | 465.0 ± 70.9 |  | R54D12 | 3 | 480.0 ± 105.0 |
| R19H12 | 3 | 480.0 ± 156.1 |  | R26F01 | 3 | 450.0 ± 292.0 |  | R54H08 | 12 | 487.5 ± 121.3 |
| R20A10 | 3 | 435.0 ± 102.1 |  | R28A09 | 3 | 450.0 ± 91.7 |  | R55A10 | 3 | 450.0 ± 82.6 |
| R20E05 | 8 | 487.5 ± 75.6 |  | R28G02 | 3 | 465.0 ± 65.4 |  | R55B01 | 16 | 457.5 ± 123.9 |
| R20E06 | 8 | 465.0 ± 76.4 |  | R29D08 | 3 | 465.0 ± 151.0 |  | R55D02 | 17 | 435.0 ± 166.8 |
| R20G12 | 12 | 435.0 ± 161.3 |  | R29E02 | 9 | 465.0 ± 109.4 |  | R55D11 | 14 | 450.0 ± 78.0 |
| R21D09 | 10 | 480.0 ± 67.7 |  | R29G11 | 3 | 390.0 ± 108.2 |  | R55D12 | 3 | 465.0 ± 176.8 |
| R21E02 | 5 | 480.0 ± 64.0 |  | R30A09 | 6 | 465.0 ± 179.6 |  | R55H04 | 11 | 390.0 ± 115.3 |
| R21E07 | 4 | 405.0 ± 7.5 |  | R30C03 | 5 | 375.0 ± 101.7 |  | R55H05 | 6 | 480.0 ± 83.6 |
| R21E08 | 4 | 457.5 ± 98.3 |  | R30F09 | 6 | 232.5 ± 211.9 |  | R56H10 | 11 | 480.0 ± 64.1 |
| R21E11 | 4 | 480.0 ± 88.7 |  | R30H05 | 3 | 450.0 ± 87.9 |  | R57C10 | 6 | 337.5 ± 180.7 |
| **Line name** | **n** | **TimeToMax[s]** |  | **Line name** | **n** | **TimeToMax[s]** |  | **Line name** | **n** | **TimeToMax[s]** |
| R21F10 | 4 | 390.0 ± 140.9 |  | R31B12 | 5 | 450.0 ± 181.7 |  | R57E06 | 3 | 375.0 ± 136.1 |
| R21G09 | 6 | 487.5 ± 158.7 |  | R31G04 | 3 | 420.0 ± 116.5 |  | R57G11 | 9 | 420.0 ± 154.9 |
| R21G12 | 3 | 450.0 ± 54.1 |  | R32C04 | 3 | 450.0 ± 106.4 |  | R59B10 | 3 | 480.0 ± 70.9 |
| R59G03 | 12 | 480.0 ± 97.9 |  | R69C07 | 3 | 465.0 ± 79.4 |  | R81B10 | 3 | 420.0 ± 91.2 |
| R59G08 | 9 | 465.0 ± 153.0 |  | R69F08 | 3 | 465.0 ± 105.4 |  | R81B12 | 6 | 465.0 ± 170.1 |
| R60A10 | 6 | 457.5 ± 220.0 |  | R70F07 | 15 | 465.0 ± 150.8 |  | R82C09 | 11 | 480.0 ± 141.6 |
| R60B09 | 6 | 480.0 ± 111.7 |  | R70F10 | 6 | 412.5 ± 156.3 |  | R82E12 | 14 | 480.0 ± 80.4 |
| R61E08 | 3 | 450.0 ± 48.2 |  | R71G03 | 3 | 375.0 ± 159.0 |  | R84B02 | 14 | 465.0 ± 137.2 |
| R61H06 | 6 | 480.0 ± 112.5 |  | R72H11 | 9 | 480.0 ± 121.5 |  | R85B06 | 11 | 465.0 ± 66.2 |
| R64C04 | 6 | 480.0 ± 122.4 |  | R73H09 | 9 | 450.0 ± 124.3 |  | R85B10 | 5 | 480.0 ± 69.9 |
| R64G09 | 6 | 487.5 ± 49.7 |  | R74B06 | 12 | 427.5 ± 130.8 |  | R86D02 | 6 | 487.5 ± 79.8 |
| R64H04 | 13 | 480.0 ± 79.9 |  | R74G10 | 3 | 435.0 ± 121.2 |  | R86G07 | 6 | 480.0 ± 57.4 |
| R65B04 | 9 | 465.0 ± 75.2 |  | R75C10 | 8 | 457.5 ± 42.2 |  | R86H12 | 4 | 480.0 ± 77.8 |
| R65C03 | 15 | 465.0 ± 89.6 |  | R75G02 | 6 | 420.0 ± 149.7 |  | R87F10 | 3 | 480.0 ± 62.4 |
| R65D06 | 18 | 480.0 ± 138.0 |  | R76B01 | 6 | 420.0 ± 127.9 |  | R87H11 | 14 | 472.5 ± 111.2 |
| R66A06 | 3 | 465.0 ± 75.0 |  | R77H03 | 5 | 435.0 ± 74.7 |  | R89E07 | 6 | 427.5 ± 131.0 |
| R67B03 | 3 | 465.0 ± 56.8 |  | R78B07 | 12 | 480.0 ± 91.4 |  | R91D09 | 6 | 405.0 ± 194.8 |
| R67E12 | 15 | 480.0 ± 85.9 |  | R78D12 | 12 | 487.5 ± 101.9 |  | R92B11 | 3 | 450.0 ± 91.7 |
| R67F01 | 3 | 465.0 ± 31.2 |  | R78E12 | 12 | 450.0 ± 94.9 |  | R92C05 | 3 | 465.0 ± 91.7 |
| R68A10 | 14 | 487.5 ± 73.9 |  | R78F01 | 6 | 397.5 ± 118.7 |  | R92D09 | 6 | 457.5 ± 165.8 |
| R68A11 | 18 | 457.5 ± 108.7 |  | R79C09 | 3 | 465.0 ± 82.6 |  | R94A07 | 6 | 487.5 ± 65.3 |
| R68C09 | 6 | 420.0 ± 92.3 |  | R79E02 | 9 | 270.0 ± 141.9 |  | R94G05 | 7 | 435.0 ± 86.6 |
| R69C04 | 12 | 487.5 ± 98.5 |  | R80B02 | 14 | 472.5 ± 117.0 |  |  |  |  |

**Supplementary Table 3.** **List of lines with altered walking activity in the ring-gap experiments of Figure 1 as shown in the evaluation of Supplementary Fig. S1b.** Lines with increased walking activity beyond the empirically determined orange line are listed here.

| **Line name** | **n** | **MaxMeanDist** | **Walked Dist.** |  | **Line name** | **n** | **MaxMeanDist** | **Walked Dist.** |
| --- | --- | --- | --- | --- | --- | --- | --- | --- |
| R09B08 | 5 | 0.558 ± 0.081 | 3.054 ± 0.857 |  | R23B07 | 3 | 0.486 ± 0.076 | 4.148 ± 0.916 |
| R10A07 | 17 | 0.576 ± 0.122 | 4.404 ± 1.781 |  | R23C02 | 9 | 0.587 ± 0.073 | 2.610 ± 0.621 |
| R10B03 | 3 | 0.612 ± 0.039 | 4.061 ± 0.710 |  | R23C04 | 4 | 0.654 ± 0.093 | 5.009 ± 1.041 |
| R10B09 | 15 | 0.585 ± 0.157 | 2.951 ± 1.406 |  | R23C08 | 5 | 0.628 ± 0.067 | 3.927 ± 1.252 |
| R10C01 | 15 | 0.493 ± 0.179 | 2.099 ± 1.453 |  | R23D11 | 3 | 0.770 ± 0.086 | 4.706 ± 1.482 |
| R10C02 | 3 | 0.627 ± 0.065 | 8.783 ± 0.896 |  | R23D12 | 6 | 0.634 ± 0.155 | 3.144 ± 1.525 |
| R10C05 | 15 | 0.565 ± 0.196 | 3.100 ± 1.646 |  | R23E03 | 3 | 0.773 ± 0.055 | 5.135 ± 0.595 |
| R10C09 | 7 | 0.709 ± 0.074 | 3.569 ± 1.160 |  | R23E04 | 6 | 0.621 ± 0.063 | 4.125 ± 1.769 |
| R10E07 | 12 | 0.534 ± 0.225 | 2.789 ± 1.429 |  | R23E06 | 3 | 0.593 ± 0.069 | 4.383 ± 1.249 |
| R10E08 | 9 | 0.654 ± 0.134 | 3.395 ± 1.274 |  | R23E09 | 3 | 0.570 ± 0.113 | 3.396 ± 0.945 |
| R10F08 | 14 | 0.541 ± 0.151 | 3.085 ± 1.574 |  | R23F01 | 3 | 0.586 ± 0.021 | 4.027 ± 0.293 |
| R10H10 | 13 | 0.538 ± 0.178 | 2.261 ± 1.519 |  | R23F03 | 3 | 0.555 ± 0.093 | 3.938 ± 0.428 |
| R11A01 | 10 | 0.597 ± 0.104 | 3.058 ± 1.385 |  | R23F04 | 3 | 0.573 ± 0.057 | 4.729 ± 0.474 |
| R11D01 | 3 | 0.528 ± 0.164 | 2.816 ± 1.348 |  | R23F05 | 6 | 0.692 ± 0.130 | 4.239 ± 0.902 |
| R11D09 | 13 | 0.582 ± 0.113 | 2.821 ± 0.969 |  | R23F09 | 3 | 0.609 ± 0.227 | 4.125 ± 1.884 |
| R12B10 | 12 | 0.484 ± 0.122 | 2.114 ± 1.714 |  | R23G02 | 3 | 0.669 ± 0.077 | 3.607 ± 1.222 |
| R12C01 | 7 | 0.716 ± 0.042 | 4.433 ± 0.808 |  | R23G04 | 3 | 0.607 ± 0.060 | 3.685 ± 0.124 |
| R12C02 | 3 | 0.484 ± 0.055 | 4.809 ± 0.895 |  | R23G05 | 3 | 0.653 ± 0.112 | 5.324 ± 3.056 |
| R12E07 | 3 | 0.509 ± 0.061 | 3.623 ± 0.793 |  | R23G07 | 3 | 0.638 ± 0.206 | 4.068 ± 1.226 |
| R13B07 | 7 | 0.331 ± 0.071 | 1.264 ± 0.176 |  | R23G10 | 3 | 0.388 ± 0.171 | 2.604 ± 2.138 |
| R13C04 | 9 | 0.590 ± 0.254 | 3.014 ± 1.972 |  | R23G11 | 3 | 0.377 ± 0.064 | 1.592 ± 1.094 |
| R13D12 | 5 | 0.265 ± 0.119 | 1.381 ± 0.415 |  | R23G12 | 11 | 0.638 ± 0.080 | 3.085 ± 0.905 |
| R13F02 | 9 | 0.443 ± 0.135 | 2.338 ± 0.868 |  | R23H02 | 3 | 0.650 ± 0.087 | 3.325 ± 0.952 |
| R13F07 | 6 | 0.491 ± 0.069 | 4.155 ± 3.027 |  | R23H09 | 3 | 0.647 ± 0.084 | 3.869 ± 1.215 |
| R13G05 | 6 | 0.502 ± 0.164 | 2.524 ± 1.400 |  | R23H10 | 3 | 0.521 ± 0.135 | 2.997 ± 0.930 |
| R14A04 | 3 | 0.460 ± 0.068 | 3.146 ± 0.490 |  | R23H12 | 3 | 0.615 ± 0.042 | 4.775 ± 0.922 |
| R14C06 | 3 | 0.535 ± 0.022 | 3.655 ± 0.800 |  | R24B08 | 4 | 0.662 ± 0.093 | 3.937 ± 1.712 |
| R14C08 | 6 | 0.375 ± 0.152 | 1.521 ± 0.891 |  | R24C01 | 3 | 0.649 ± 0.026 | 3.720 ± 0.566 |
| R14D12 | 3 | 0.726 ± 0.077 | 3.700 ± 0.731 |  | R24D01 | 3 | 0.676 ± 0.046 | 3.832 ± 0.971 |
| R14E09 | 14 | 0.579 ± 0.086 | 2.555 ± 1.287 |  | R24D04 | 3 | 0.439 ± 0.056 | 2.135 ± 0.927 |
| R14F05 | 6 | 0.634 ± 0.165 | 2.969 ± 1.521 |  | R24D08 | 3 | 0.671 ± 0.072 | 4.097 ± 0.970 |
| R14H09 | 3 | 0.634 ± 0.034 | 4.033 ± 1.063 |  | R24D12 | 5 | 0.706 ± 0.228 | 3.516 ± 1.830 |
| R15C05 | 3 | 0.389 ± 0.030 | 1.742 ± 0.470 |  | R24E01 | 3 | 0.764 ± 0.054 | 4.045 ± 0.941 |
| R15D07 | 3 | 0.481 ± 0.056 | 2.679 ± 0.259 |  | R24E02 | 3 | 0.482 ± 0.032 | 3.580 ± 1.277 |
| R15F08 | 11 | 0.484 ± 0.131 | 2.073 ± 0.974 |  | R24E03 | 3 | 0.539 ± 0.092 | 3.036 ± 1.448 |
| R15H01 | 3 | 0.616 ± 0.035 | 3.127 ± 1.964 |  | R24E04 | 3 | 0.510 ± 0.091 | 3.106 ± 0.297 |
| R16A05 | 3 | 0.730 ± 0.030 | 4.753 ± 0.382 |  | R24E06 | 4 | 0.628 ± 0.097 | 3.487 ± 1.062 |
| R16A06 | 6 | 0.568 ± 0.179 | 3.009 ± 1.594 |  | R24E10 | 3 | 0.641 ± 0.054 | 3.762 ± 0.455 |
| R16E05 | 3 | 0.755 ± 0.048 | 4.530 ± 0.261 |  | R24E12 | 6 | 0.514 ± 0.210 | 2.537 ± 1.064 |
| R16F02 | 6 | 0.420 ± 0.099 | 2.399 ± 0.933 |  | R24F01 | 3 | 0.595 ± 0.049 | 3.425 ± 1.143 |
| R16F05 | 7 | 0.284 ± 0.096 | 1.366 ± 0.406 |  | R24F04 | 3 | 0.634 ± 0.104 | 3.546 ± 0.998 |
| R17C08 | 3 | 0.367 ± 0.212 | 1.506 ± 0.545 |  | R24F05 | 3 | 0.549 ± 0.022 | 2.679 ± 0.346 |
| R17D02 | 12 | 0.579 ± 0.152 | 3.323 ± 1.553 |  | R24F07 | 3 | 0.395 ± 0.103 | 1.817 ± 0.251 |
| R17D06 | 6 | 0.492 ± 0.079 | 2.209 ± 0.594 |  | R24F10 | 6 | 0.314 ± 0.135 | 1.642 ± 1.681 |
| **Line name** | **n** | **MaxMeanDist** | **Walked Dist.** |  | **Line name** | **n** | **MaxMeanDist** | **Walked Dist.** |
| R17D11 | 6 | 0.665 ± 0.074 | 3.532 ± 0.554 |  | R24F12 | 3 | 0.567 ± 0.151 | 3.430 ± 0.676 |
| R17D12 | 8 | 0.548 ± 0.166 | 3.958 ± 1.034 |  | R24G05 | 3 | 0.676 ± 0.055 | 3.474 ± 1.365 |
| R17E04 | 3 | 0.634 ± 0.018 | 2.965 ± 0.197 |  | R25A05 | 3 | 0.521 ± 0.056 | 2.130 ± 0.627 |
| R17F04 | 3 | 0.683 ± 0.064 | 3.335 ± 1.018 |  | R25A06 | 3 | 0.361 ± 0.033 | 1.793 ± 0.564 |
| R17F06 | 3 | 0.611 ± 0.152 | 2.952 ± 1.306 |  | R25A07 | 3 | 0.585 ± 0.100 | 2.697 ± 0.497 |
| R17F07 | 3 | 0.591 ± 0.027 | 2.920 ± 0.220 |  | R25B04 | 3 | 0.736 ± 0.016 | 3.772 ± 0.661 |
| R17F08 | 3 | 0.614 ± 0.043 | 3.309 ± 0.997 |  | R25B05 | 6 | 0.443 ± 0.073 | 3.780 ± 1.229 |
| R17G02 | 3 | 0.629 ± 0.070 | 3.744 ± 0.316 |  | R25B08 | 3 | 0.581 ± 0.095 | 4.020 ± 0.316 |
| R17G09 | 3 | 0.490 ± 0.129 | 2.140 ± 0.324 |  | R25B11 | 3 | 0.578 ± 0.023 | 3.383 ± 0.530 |
| R17G11 | 3 | 0.511 ± 0.106 | 3.151 ± 0.297 |  | R25C03 | 6 | 0.671 ± 0.153 | 3.188 ± 0.600 |
| R17G12 | 4 | 0.633 ± 0.033 | 2.938 ± 1.047 |  | R25C07 | 3 | 0.626 ± 0.098 | 3.312 ± 1.535 |
| R17H06 | 3 | 0.620 ± 0.082 | 3.221 ± 1.281 |  | R25C10 | 3 | 0.652 ± 0.075 | 4.369 ± 1.481 |
| R17H08 | 3 | 0.588 ± 0.050 | 3.116 ± 1.294 |  | R25C12 | 3 | 0.702 ± 0.065 | 4.065 ± 1.243 |
| R18A01 | 5 | 0.578 ± 0.257 | 2.560 ± 1.537 |  | R25D02 | 3 | 0.620 ± 0.099 | 3.221 ± 0.768 |
| R18A02 | 3 | 0.593 ± 0.144 | 3.277 ± 0.137 |  | R25D07 | 3 | 0.676 ± 0.054 | 4.647 ± 1.558 |
| R18A06 | 3 | 0.647 ± 0.078 | 3.617 ± 0.450 |  | R25D09 | 3 | 0.686 ± 0.079 | 3.966 ± 1.422 |
| R18A09 | 3 | 0.420 ± 0.058 | 3.263 ± 0.993 |  | R25F11 | 17 | 0.500 ± 0.163 | 1.987 ± 1.368 |
| R18A10 | 3 | 0.607 ± 0.073 | 3.078 ± 0.861 |  | R25G04 | 12 | 0.673 ± 0.061 | 3.394 ± 1.650 |
| R18A11 | 12 | 0.565 ± 0.059 | 2.751 ± 1.094 |  | R25H10 | 3 | 0.753 ± 0.060 | 5.943 ± 0.748 |
| R18B01 | 3 | 0.511 ± 0.259 | 2.953 ± 0.291 |  | R26A02 | 6 | 0.710 ± 0.152 | 3.838 ± 2.449 |
| R18B04 | 3 | 0.492 ± 0.078 | 2.088 ± 0.812 |  | R26C03 | 12 | 0.520 ± 0.175 | 2.421 ± 1.311 |
| R18B08 | 3 | 0.732 ± 0.042 | 4.274 ± 1.146 |  | R26E06 | 3 | 0.560 ± 0.032 | 2.419 ± 0.539 |
| R18B09 | 3 | 0.528 ± 0.078 | 2.514 ± 0.328 |  | R26G11 | 3 | 0.477 ± 0.021 | 2.521 ± 0.864 |
| R18B11 | 12 | 0.554 ± 0.137 | 2.618 ± 1.687 |  | R27A08 | 7 | 0.648 ± 0.058 | 3.544 ± 0.973 |
| R18C01 | 3 | 0.484 ± 0.105 | 3.815 ± 0.648 |  | R28F08 | 3 | 0.663 ± 0.040 | 4.932 ± 0.510 |
| R18C02 | 3 | 0.607 ± 0.092 | 2.723 ± 0.699 |  | R29C06 | 14 | 0.547 ± 0.134 | 3.134 ± 1.796 |
| R18C03 | 3 | 0.681 ± 0.093 | 3.665 ± 1.377 |  | R29E10 | 15 | 0.650 ± 0.102 | 3.575 ± 1.263 |
| R18C05 | 3 | 0.592 ± 0.149 | 3.070 ± 0.788 |  | R29F12 | 14 | 0.568 ± 0.082 | 2.575 ± 1.354 |
| R18C08 | 3 | 0.639 ± 0.037 | 2.932 ± 0.723 |  | R30D06 | 9 | 0.464 ± 0.055 | 2.898 ± 0.764 |
| R18C11 | 12 | 0.610 ± 0.087 | 3.049 ± 1.207 |  | R30D11 | 9 | 0.630 ± 0.065 | 3.069 ± 0.928 |
| R18D02 | 3 | 0.624 ± 0.084 | 2.972 ± 0.857 |  | R32D05 | 3 | 0.377 ± 0.147 | 1.728 ± 0.425 |
| R18D06 | 3 | 0.513 ± 0.110 | 3.125 ± 1.561 |  | R34A03 | 3 | 0.388 ± 0.088 | 1.595 ± 0.069 |
| R19A01 | 3 | 0.546 ± 0.013 | 5.474 ± 0.787 |  | R34C03 | 3 | 0.171 ± 0.043 | 0.971 ± 0.267 |
| R19B06 | 6 | 0.629 ± 0.090 | 5.311 ± 1.482 |  | R34E11 | 3 | 0.286 ± 0.048 | 4.265 ± 0.354 |
| R19C10 | 3 | 0.533 ± 0.072 | 2.683 ± 0.203 |  | R34G07 | 12 | 0.595 ± 0.092 | 2.958 ± 1.468 |
| R19D05 | 4 | 0.658 ± 0.094 | 4.438 ± 0.687 |  | R34H12 | 15 | 0.617 ± 0.108 | 2.955 ± 1.253 |
| R19G01 | 12 | 0.688 ± 0.137 | 4.200 ± 1.908 |  | R35C09 | 6 | 0.341 ± 0.049 | 1.699 ± 0.505 |
| R20A06 | 6 | 0.599 ± 0.148 | 2.794 ± 1.410 |  | R37A02 | 3 | 0.455 ± 0.074 | 1.756 ± 0.483 |
| R20A07 | 6 | 0.725 ± 0.047 | 4.210 ± 0.459 |  | R38A07 | 12 | 0.585 ± 0.105 | 3.381 ± 1.308 |
| R20A10 | 3 | 0.784 ± 0.082 | 4.917 ± 1.386 |  | R38B08 | 3 | 0.666 ± 0.037 | 3.265 ± 0.692 |
| R20B02 | 4 | 0.806 ± 0.088 | 5.103 ± 1.127 |  | R39G09 | 6 | 0.441 ± 0.045 | 1.964 ± 0.489 |
| R20B05 | 7 | 0.603 ± 0.148 | 3.392 ± 1.453 |  | R40A06 | 8 | 0.626 ± 0.028 | 3.482 ± 1.478 |
| R20C01 | 3 | 0.502 ± 0.086 | 2.431 ± 0.370 |  | R41A10 | 9 | 0.624 ± 0.081 | 3.952 ± 0.784 |
| R20D11 | 6 | 0.602 ± 0.032 | 5.392 ± 1.139 |  | R41G08 | 15 | 0.599 ± 0.125 | 2.925 ± 1.802 |
| R20E03 | 15 | 0.737 ± 0.096 | 3.968 ± 1.799 |  | R42C01 | 9 | 0.705 ± 0.065 | 4.750 ± 0.621 |
| R20E07 | 6 | 0.665 ± 0.078 | 5.501 ± 1.728 |  | R42G10 | 6 | 0.272 ± 0.084 | 1.301 ± 0.384 |
| R20E12 | 11 | 0.501 ± 0.131 | 2.592 ± 1.671 |  | R43E05 | 12 | 0.624 ± 0.059 | 2.995 ± 1.561 |
| R20F10 | 10 | 0.578 ± 0.072 | 6.595 ± 2.161 |  | R43G06 | 9 | 0.633 ± 0.079 | 3.950 ± 0.924 |
| R20F11 | 5 | 0.677 ± 0.036 | 3.287 ± 2.674 |  | R44C02 | 5 | 0.602 ± 0.073 | 4.238 ± 0.989 |
| R20G01 | 6 | 0.660 ± 0.129 | 3.839 ± 1.250 |  | R44F09 | 9 | 0.544 ± 0.076 | 3.176 ± 1.180 |
| R20G12 | 12 | 0.403 ± 0.062 | 1.545 ± 0.564 |  | R45D04 | 10 | 0.615 ± 0.160 | 3.311 ± 1.025 |
| R20H10 | 3 | 0.594 ± 0.050 | 3.646 ± 0.845 |  | R45E04 | 6 | 0.710 ± 0.111 | 3.863 ± 0.995 |
| R21A03 | 3 | 0.767 ± 0.093 | 4.094 ± 0.374 |  | R46A08 | 8 | 0.552 ± 0.094 | 4.771 ± 1.833 |
| R21A04 | 15 | 0.582 ± 0.194 | 2.493 ± 1.625 |  | R46B05 | 10 | 0.510 ± 0.050 | 2.226 ± 1.015 |
| **Line name** | **n** | **MaxMeanDist** | **Walked Dist.** |  | **Line name** | **n** | **MaxMeanDist** | **Walked Dist.** |
| R21A05 | 3 | 0.635 ± 0.061 | 4.242 ± 0.267 |  | R47H08 | 13 | 0.459 ± 0.104 | 2.999 ± 1.479 |
| R21A09 | 5 | 0.637 ± 0.076 | 4.850 ± 1.142 |  | R48G08 | 6 | 0.444 ± 0.037 | 1.972 ± 0.481 |
| R21B04 | 8 | 0.637 ± 0.095 | 4.160 ± 1.699 |  | R49C03 | 3 | 0.408 ± 0.067 | 1.610 ± 0.326 |
| R21B06 | 6 | 0.622 ± 0.053 | 4.900 ± 1.156 |  | R49F09 | 17 | 0.762 ± 0.111 | 4.403 ± 1.670 |
| R21E05 | 3 | 0.553 ± 0.019 | 3.940 ± 1.086 |  | R50A08 | 17 | 0.597 ± 0.135 | 2.994 ± 1.139 |
| R21F02 | 3 | 0.667 ± 0.092 | 3.475 ± 1.085 |  | R50B02 | 10 | 0.656 ± 0.074 | 4.217 ± 1.487 |
| R21F10 | 4 | 0.724 ± 0.044 | 4.082 ± 0.845 |  | R50E01 | 10 | 0.661 ± 0.072 | 3.673 ± 1.341 |
| R21F11 | 3 | 0.682 ± 0.056 | 3.665 ± 0.587 |  | R50F04 | 12 | 0.702 ± 0.091 | 3.482 ± 1.382 |
| R21G02 | 3 | 0.642 ± 0.022 | 3.625 ± 0.713 |  | R50G11 | 15 | 0.675 ± 0.131 | 3.271 ± 1.503 |
| R21G05 | 3 | 0.692 ± 0.007 | 3.385 ± 0.218 |  | R51C07 | 17 | 0.697 ± 0.103 | 3.432 ± 1.485 |
| R21G12 | 3 | 0.639 ± 0.109 | 3.135 ± 0.883 |  | R52A01 | 3 | 0.527 ± 0.087 | 2.386 ± 0.701 |
| R21H03 | 3 | 0.643 ± 0.115 | 3.133 ± 0.873 |  | R53D12 | 6 | 0.284 ± 0.052 | 1.344 ± 0.977 |
| R21H05 | 3 | 0.631 ± 0.013 | 3.639 ± 0.172 |  | R54B05 | 7 | 0.636 ± 0.074 | 3.748 ± 0.752 |
| R21H07 | 3 | 0.576 ± 0.094 | 2.763 ± 1.239 |  | R54G07 | 15 | 0.625 ± 0.105 | 3.116 ± 1.783 |
| R21H08 | 3 | 0.677 ± 0.032 | 4.341 ± 0.266 |  | R55C11 | 20 | 0.520 ± 0.105 | 2.668 ± 1.242 |
| R21H10 | 9 | 0.674 ± 0.111 | 3.760 ± 1.800 |  | R55D05 | 6 | 0.364 ± 0.049 | 2.080 ± 0.578 |
| R22A05 | 3 | 0.651 ± 0.006 | 5.566 ± 1.131 |  | R55D12 | 3 | 0.628 ± 0.074 | 3.573 ± 1.991 |
| R22A06 | 3 | 0.757 ± 0.054 | 4.265 ± 0.735 |  | R57C08 | 9 | 0.524 ± 0.134 | 3.319 ± 0.645 |
| R22A09 | 6 | 0.761 ± 0.077 | 4.272 ± 0.909 |  | R59F09 | 10 | 0.444 ± 0.197 | 2.870 ± 1.013 |
| R22A11 | 4 | 0.763 ± 0.030 | 4.273 ± 0.601 |  | R59G08 | 9 | 0.476 ± 0.194 | 2.306 ± 1.484 |
| R22A12 | 4 | 0.795 ± 0.063 | 4.427 ± 0.862 |  | R61C03 | 9 | 0.627 ± 0.078 | 3.224 ± 0.810 |
| R22B04 | 6 | 0.438 ± 0.277 | 1.862 ± 1.518 |  | R61C12 | 12 | 0.617 ± 0.073 | 3.472 ± 1.142 |
| R22C04 | 5 | 0.340 ± 0.358 | 1.387 ± 2.193 |  | R61H03 | 14 | 0.343 ± 0.150 | 2.847 ± 1.346 |
| R22C08 | 3 | 0.695 ± 0.049 | 3.410 ± 0.914 |  | R64D07 | 14 | 0.657 ± 0.136 | 4.615 ± 1.402 |
| R22C09 | 3 | 0.692 ± 0.029 | 5.212 ± 0.724 |  | R65B07 | 18 | 0.536 ± 0.132 | 4.069 ± 1.328 |
| R22D03 | 9 | 0.464 ± 0.214 | 1.801 ± 1.456 |  | R65B08 | 15 | 0.741 ± 0.120 | 4.444 ± 1.735 |
| R22E03 | 7 | 0.597 ± 0.113 | 2.710 ± 2.059 |  | R65B09 | 17 | 0.552 ± 0.090 | 2.730 ± 1.264 |
| R22E07 | 4 | 0.676 ± 0.096 | 5.616 ± 1.342 |  | R66B05 | 3 | 0.353 ± 0.208 | 1.380 ± 0.641 |
| R22E08 | 4 | 0.703 ± 0.081 | 3.829 ± 0.773 |  | R67B09 | 13 | 0.627 ± 0.106 | 4.037 ± 1.260 |
| R22F01 | 3 | 0.665 ± 0.061 | 3.577 ± 0.380 |  | R67D04 | 3 | 0.228 ± 0.011 | 1.372 ± 0.375 |
| R22F06 | 3 | 0.329 ± 0.079 | 1.603 ± 0.152 |  | R67E03 | 12 | 0.634 ± 0.077 | 2.875 ± 1.622 |
| R22F08 | 5 | 0.739 ± 0.000 | 4.360 ± 1.702 |  | R69H11 | 18 | 0.754 ± 0.099 | 4.060 ± 1.575 |
| R22F09 | 4 | 0.686 ± 0.092 | 3.541 ± 0.881 |  | R70B10 | 6 | 0.261 ± 0.066 | 1.338 ± 0.627 |
| R22F12 | 6 | 0.686 ± 0.046 | 4.106 ± 1.477 |  | R70G12 | 3 | 0.297 ± 0.133 | 1.372 ± 0.394 |
| R22G06 | 4 | 0.434 ± 0.054 | 2.841 ± 0.486 |  | R72E12 | 11 | 0.603 ± 0.145 | 3.154 ± 1.150 |
| R22G09 | 4 | 0.632 ± 0.082 | 3.626 ± 1.092 |  | R72F09 | 3 | 0.651 ± 0.109 | 3.532 ± 1.156 |
| R22G10 | 7 | 0.477 ± 0.137 | 2.279 ± 1.103 |  | R75B02 | 6 | 0.526 ± 0.078 | 2.154 ± 0.601 |
| R22G12 | 4 | 0.641 ± 0.120 | 4.015 ± 0.363 |  | R75F06 | 18 | 0.462 ± 0.130 | 1.881 ± 0.726 |
| R22H01 | 7 | 0.689 ± 0.143 | 3.458 ± 1.776 |  | R76E09 | 6 | 0.450 ± 0.103 | 2.024 ± 0.878 |
| R22H04 | 4 | 0.584 ± 0.074 | 2.772 ± 0.547 |  | R81E05 | 17 | 0.565 ± 0.143 | 3.029 ± 1.972 |
| R22H06 | 4 | 0.782 ± 0.068 | 4.437 ± 1.154 |  | R89A08 | 8 | 0.491 ± 0.120 | 2.062 ± 1.026 |
| R22H07 | 7 | 0.566 ± 0.163 | 2.749 ± 1.499 |  | R89B02 | 6 | 0.481 ± 0.142 | 2.174 ± 0.601 |
| R22H08 | 4 | 0.671 ± 0.068 | 4.095 ± 0.897 |  | R89D06 | 9 | 0.564 ± 0.088 | 2.803 ± 1.582 |
| R22H11 | 4 | 0.791 ± 0.056 | 5.891 ± 1.123 |  | R91A08 | 6 | 0.517 ± 0.092 | 2.170 ± 0.445 |
| R23A11 | 3 | 0.749 ± 0.033 | 4.419 ± 0.327 |  | R91F08 | 5 | 0.602 ± 0.175 | 3.336 ± 1.534 |
| R23B04 | 6 | 0.673 ± 0.172 | 4.243 ± 1.562 |  |  |  |  |  |

**Supplementary Table 4 Statistical tables** with p-values for comparisons with Wilcoxon rank sum tests and numbers of flies tested per gap width.

| **Figure 2** | **Wilcoxon rank sum tests against pBDPGAL4U; all lines drive UAS-*shi^ts^* at 34°C** | | | | | | | | | |
| --- | --- | --- | --- | --- | --- | --- | --- | --- | --- | --- |
|  | **Attempts** | | | | | | **Success** | | | |
| **Gap width** | **2.5mm** | **3.0mm** | **3.5mm** | **4.0mm** | **5.0mm** | **6.0mm** | **2.5mm** | **3.0mm** | **3.5mm** | **4.0mm** |
| **R22F08** | 0.3275 | 0.2745 | 0.2741 | 0.2806 | 9.64E-05 | 6.51E-05 | 0.6524 | 0.0764 | 0.4049 | 0.4789 |
| **R24D10** | 3.31E-05 | 5.20E-06 | 1.11E-06 | 2.03E-06 | 2.74E-06 | 0.0014 | 2.52E-05 | 4.83E-06 | 9.33E-07 | 0.0431 |
| **R23D06** | 0.4636 | 0.7775 | 0.0995 | 0.2047 | 0.6485 | 0.9514 | 0.1575 | 0.0120 | 2.66E-05 | 0.0431 |
| **R61H03** | 2.61E-04 | 3.62E-04 | 2.82E-06 | 2.02E-05 | 2.35E-05 | 2.51E-04 | 1.05E-04 | 4.56E-05 | 4.71E-06 | 0.0431 |
| **R68A11** | 0.3250 | 0.0293 | 0.3669 | 0.0045 | 0.1946 | 0.0222 | 0.2470 | 0.1539 | 0.1495 | 0.0021 |

| **Figure 2** | **Number of flies n** | | | | | |
| --- | --- | --- | --- | --- | --- | --- |
| **Gap width** | **2.5mm** | **3.0mm** | **3.5mm** | **4.0mm** | **5.0mm** | **6.0mm** |
| **pBDPGAL4U** | 23 | 21 | 24 | 41 | 34 | 32 |
| **R22F08** | 10 | 10 | 14 | 25 | 35 | 20 |
| **R24D10** | 10 | 10 | 10 | 10 | 10 | 10 |
| **R23D06** | 10 | 17 | 17 | 10 | 10 | 10 |
| **R61H03** | 10 | 10 | 10 | 10 | 10 | 10 |
| **R68A11** | 10 | 10 | 10 | 30 | 10 | 10 |

| **Figure 3** | **Wilcoxon rank sum tests against pBDPGAL4U; all lines drive UAS>*shi^ts^* at 34°C** | | | | | | | | | |
| --- | --- | --- | --- | --- | --- | --- | --- | --- | --- | --- |
| **& Fig. S2** | **Attempts** | | | | | | **Success** | | | |
| **Gap width** | **2.5mm** | **3.0mm** | **3.5mm** | **4.0mm** | **5.0mm** | **6.0mm** | **2.5mm** | **3.0mm** | **3.5mm** | **4.0mm** |
| **R22F08** | 0.3275 | 0.2745 | 0.2741 | 0.2806 | 9.64E-05 | 6.51E-05 | 0.6524 | 0.0764 | 0.4049 | 0.4789 |
| **R20C11‑R25B02** | 0.3764 | 0.2237 | 0.2528 | 0.0619 | 2.05E-04 | 0.0015 | 0.1020 | 0.0481 | 0.2635 | 0.3576 |
| **R25B02‑R48D11** | 0.4003 | 0.0715 | 0.5883 | 0.8137 | 0.0250 | 0.0065 | 0.9145 | 0.0684 | 0.4276 | 0.2131 |
| **R17C06‑R25B02** | 0.7424 | 0.5380 | 0.3793 | 0.0688 | 0.0018 | 0.0236 | 0.1954 | 0.0083 | 0.2435 | 0.2226 |
| **R26H02‑R29G11** | 0.4435 | 0.0749 | 0.3010 | 0.9910 | 0.0331 | 0.0013 | 0.0422 | 0.0015 | 0.0550 | 0.0431 |
| **R35A03‑R29G11** | 0.1946 | 0.4313 | 0.2795 | 0.2358 | 4.94E-04 | 0.0086 | 0.0034 | 6.30E-04 | 0.0020 | 0.5685 |
| **R20C11‑R48D11** | 0.9826 | 0.3187 | 0.6654 | 0.0921 | 7.51E-06 | 0.0025 | 0.0601 | 1.10E-04 | 0.0387 | 0.8266 |

| **Figure 3 & Fig. S2** | **Wilcoxon rank sum tests against R20C11‑R48D11 (C2 & C3);  all lines drive UAS>*shi^ts^* at 34°C** | | | | | | | |
| --- | --- | --- | --- | --- | --- | --- | --- | --- |
|  | **Attempts** | | | | | | **Cell type** | |
| **Gap width** | **2.5mm** | **3.0mm** | **3.5mm** | **4.0mm** | **5.0mm** | **6.0mm** |  |  |
| **R20C11‑R25B02** | 0.403 | 0.9766 | 0.9385 | 0.9547 | 0.0302 | 0.4189 | C2 |  |
| **R25B02‑R48D11** | 0.3909 | 0.5561 | 0.3025 | 0.1386 | 0.0015 | 0.4382 | C2 |  |
| **R17C06‑R25B02** | 0.8748 | 0.7577 | 0.2831 | 0.7021 | 0.0562 | 0.0724 | C2 |  |
| **R26H02‑R29G11** | 0.4816 | 0.1248 | 1.0000 | 0.2399 | 0.0012 | 0.2911 | C3 |  |
| **R35A03‑R29G11** | 0.1811 | 0.8469 | 0.2512 | 0.6418 | 0.0423 | 0.3114 | C3 |  |

| **Figure 3**  **& Fig. S2** | **Number of flies n** | | | | | | **Cell type** |
| --- | --- | --- | --- | --- | --- | --- | --- |
| **Gap width** | **2.5mm** | **3.0mm** | **3.5mm** | **4.0mm** | **5.0mm** | **6.0mm** |  |
| **pBDPGAL4U** | 23 | 21 | 24 | 41 | 34 | 32 | empty |
| **R22F08** | 10 | 10 | 14 | 25 | 35 | 20 | C2patchy |
| **R20C11‑R25B02** | 10 | 15 | 10 | 15 | 25 | 20 | C2 |
| **R25B02‑R48D11** | 10 | 10 | 10 | 19 | 25 | 14 | C2 |
| **R17C06‑R25B02** | 10 | 10 | 10 | 20 | 20 | 29 | C2 |
| **R26H02‑R29G11** | 10 | 10 | 10 | 10 | 15 | 15 | C3 |
| **R35A03‑R29G11** | 10 | 10 | 10 | 10 | 15 | 15 | C3 |
| **R20C11‑R48D11** | 10 | 10 | 10 | 10 | 15 | 15 | C2 & C3 |

| **Figure 4** | **Wilcoxon rank sum tests against pBDPGAL4U; all lines drive UAS>*dTrpA1* at 29°C** | | | | | | | | | |
| --- | --- | --- | --- | --- | --- | --- | --- | --- | --- | --- |
| **& Fig. S3** | **Attempts** | | | | | | **Success** | | | |
| **Gap width** | **2.5mm** | **3.0mm** | **3.5mm** | **4.0mm** | **5.0mm** | **6.0mm** | **2.5mm** | **3.0mm** | **3.5mm** | **4.0mm** |
| **R20C11‑R25B02** | 7.96E-05 | 0.0229 | 0.0146 | 0.1002 | 0.3702 | 0.2211 | 4.94E-05 | 0.0082 | 0.0117 | 1.0000 |
| **R25B02‑R48D11** | 0.0024 | 0.0024 | 0.0016 | 0.0540 | 0.5630 | 0.1226 | 3.95E-04 | 3.88E-04 | 2.06E-04 | 0.8079 |
| **R17C06‑R25B02** | 0.0032 | 0.1409 | 0.0015 | 0.0858 | 0.9743 | 0.0403 | 0.0075 | 0.1642 | 7.57E-04 | 0.1098 |
| **R26H02‑R29G11** | 0.0063 | 0.0030 | 0.0064 | 0.1873 | 0.1664 | 0.3022 | 0.0205 | 0.0060 | 0.0035 | 0.1572 |
| **R35A03‑R29G11** | 0.0023 | 3.81E-04 | 0.0036 | 0.0010 | 0.0446 | 0.2302 | 0.0034 | 1.56E-04 | 0.0015 | 0.0123 |
| **R20C11‑R48D11** | 0.0013 | 0.0325 | 0.0074 | 0.0161 | 0.3260 | 0.3656 | 0.0013 | 0.0214 | 0.0091 | 0.0549 |

| **Figure 4**  **& Fig. S3** | **Number of flies n** | | | | | | **Cell type** |
| --- | --- | --- | --- | --- | --- | --- | --- |
| **Gap width** | **2.5mm** | **3.0mm** | **3.5mm** | **4.0mm** | **5.0mm** | **6.0mm** |  |
| **pBDPGAL4U** | 24 | 17 | 26 | 15 | 13 | 12 | empty |
| **R20C11‑R25B02** | 14 | 25 | 25 | 14 | 10 | 10 | C2 |
| **R25B02‑R48D11** | 13 | 20 | 20 | 13 | 10 | 10 | C2 |
| **R17C06‑R25B02** | 10 | 10 | 10 | 10 | 10 | 10 | C2 |
| **R26H02‑R29G11** | 10 | 10 | 10 | 10 | 10 | 10 | C3 |
| **R35A03‑R29G11** | 20 | 17 | 20 | 15 | 11 | 10 | C3 |
| **R20C11‑R48D11** | 10 | 15 | 10 | 10 | 10 | 10 | C2 & C3 |

| **Fig. S4** | **Wilcoxon rank sum tests against pBDPGAL4U>*dTrpA1* at 29°C** | | | | | | | | | |
| --- | --- | --- | --- | --- | --- | --- | --- | --- | --- | --- |
|  | **Attempts** | | | | | | **Success** | | | |
| **Gap width** | **2.5mm** | **3.0mm** | **3.5mm** | **4.0mm** | **5.0mm** | **6.0mm** | **2.5mm** | **3.0mm** | **3.5mm** | **4.0mm** |
| **pBDPGAL4U >*shi^ts^* 34°C** | 0.7627 | 0.7570 | 0.3433 | 0.2660 | 0.7147 | 0.9243 | 0.9265 | 0.9252 | 0.0030 | 0.0412 |
| **pBDPGAL4U at >*Trp* 20°C** | 0.9694 | 0.9189 | 0.2424 | 0.0090 | 1.0000 | 0.0677 | 0.7444 | 0.8183 | 0.0484 | 0.0234 |
| **R20C11‑R25B02 >*Trp* 27°C** | 0.1518 | 0.3875 | 0.3547 | 0.2595 | 0.0971 | 0.1462 | 0.0339 | 0.2311 | 0.0884 | 0.5243 |
| **R25B02‑R48D11 >*Trp* 27°C** | 0.6303 | 0.1202 | 0.0040 | 0.8881 | 0.8983 | 0.1666 | 0.0845 | 0.0553 | 0.0016 | 0.0446 |

| **Fig. S4** | **Wilcoxon rank sum tests against pBDPGAL4U>*dTrpA1* at 20°C** | | | | | | | | | |
| --- | --- | --- | --- | --- | --- | --- | --- | --- | --- | --- |
|  | **Attempts** | | | | | | **Success** | | | |
| **Gap width** | **2.5mm** | **3.0mm** | **3.5mm** | **4.0mm** | **5.0mm** | **6.0mm** | **2.5mm** | **3.0mm** | **3.5mm** | **4.0mm** |
| **R20C11‑R25B02 >*Trp* 20°C** | 1.0000 | 0.0299 | 0.4786 | 0.5083 | 0.1315 | 0.4771 | 0.9692 | 0.0102 | 0.2498 | 0.3681 |

| **Fig. S4** | **Number of flies n** | | | | | | **Cell type** | **Temp** |
| --- | --- | --- | --- | --- | --- | --- | --- | --- |
| **Gap width** | **2.5mm** | **3.0mm** | **3.5mm** | **4.0mm** | **5.0mm** | **6.0mm** |  |  |
| **pBDPGAL4U>*shi*** | 23 | 21 | 24 | 41 | 34 | 32 | empty | 29°C |
| **pBDPGAL4U>*Trp*** | 24 | 17 | 26 | 15 | 13 | 12 | empty | 29°C |
| **pBDPGAL4U>*Trp*** | 10 | 10 | 10 | 10 | 10 | 10 | empty | 20°C |
| **R20C11‑R25B02** | 10 | 10 | 10 | 10 | 15 | 10 | C2 | 27°C |
| **R25B02‑R48D11** | 10 | 10 | 10 | 12 | 10 | 10 | C2 | 27°C |
| **R20C11‑R25B02** | 10 | 10 | 10 | 10 | 10 | 10 | C2 | 20°C |

| **Figure 5** | **Wilcoxon rank sum tests; driver line R20C11-R25B02** | | | | | |
| --- | --- | --- | --- | --- | --- | --- |
| **Gap type** | **Normal dark** | | **Transparent** | | **Vertical stripes** | |
| **Effector** | **>*shi^ts^*** | **>*dTrpA1*** | **>*shi^ts^*** | **>*dTrpA1*** | **>*shi^ts^*** | **>*dTrpA1*** |
| **Normal / >*shi^ts^*** | **----------** |  |  | |  | |
| **Normal / >*TrpA1*** | 0.0077 | **----------** |  |  |  |  |
| **Transp. / >*shi^ts^*** | 1.48E-04 | | **----------** |  |  | |
| **Transp. / >*TrpA1*** |  |  | 0.3109 | **----------** |  |  |
| **Striped / *shi^ts^*** | 0.0330 | | 3.93E-06 | | **----------** |  |
| **Striped / *TrpA1*** |  |  |  |  | 0.0406 | **----------** |

| **Figure 5** | **Number of flies n** | | | | | |
| --- | --- | --- | --- | --- | --- | --- |
| **Gap type** | **Normal dark** | | **Transparent** | | **Vertical stripes** | |
| **Effector** | **>*shi^ts^*** | **>*dTrpA1*** | **>*shi^ts^*** | **>*dTrpA1*** | **>*shi^ts^*** | **>*dTrpA1*** |
| **R20C11-R25B02** | 15 | 14 | 10 | 10 | 10 | 10 |

**Supplementary Table 5 p-values of Wilcoxon rank sum tests** asking whether C3>*shi^ts^* flies and C2+C3>*shi^ts^* flies are less successful than C2>*shi^ts^* flies when attempting to overcome surmountable gaps; likewise whether C2+C3>*shi^ts^* flies are less successful than C3>*shi^ts^* flies.

| **Line** | **vs. Line** | **Gap 2.5mm** | **Gap 3.0mm** | **Gap 3.5mm** | **Gap 4.0mm** | **Data set  shown in** |
| --- | --- | --- | --- | --- | --- | --- |
| C2_R20C11-R25B02 | C3_R35A03-R29G11 | 0.0440 | 0.0148 | 0.0014 | 0.2797 | Fig.3 – Fig.3 |
| C2_R20C11-R25B02 | C3_R26H02-R29G11 | 0.5569 | 0.0240 | 0.0067 | 0.0146 | Fig.3 – Fig.S2 |
| C2_R17C06-R25B02 | C3_R35A03-R29G11 | 0.0430 | 0.3316 | 0.1114 | 0.2491 | Fig.S2 – Fig.3 |
| C2_R17C06-R25B02 | C3_R26H02-R29G11 | 0.3899 | 0.6670 | 0.4608 | 0.0153 | Fig.S2 – Fig.S2 |
| C2_R25B02-R48D11 | C3_R35A03-R29G11 | 0.0171 | 0.0205 | 0.0417 | 0.7536 | Fig.S2 – Fig.3 |
| C2_R25B02-R48D11 | C3_R26H02-R29G11 | 0.1435 | 0.0542 | 0.3959 | 0.2081 | Fig.S2 – Fig.S2 |
|  |  |  |  |  |  |  |
| C2_R20C11-R25B02 | C2+C3_R20C11-R48D11 | 0.4376 | 0.0012 | 0.0359 | 0.3835 | Fig.3 – Fig.3 |
| C2_R17C06-R25B02 | C2+C3_R20C11-R48D11 | 0.4404 | 0.0897 | 0.6717 | 0.3024 | Fig.S2 – Fig.3 |
| C2_R25B02-R48D11 | C2+C3_R20C11-R48D11 | 0.1402 | 0.0029 | 0.3538 | 0.4540 | Fig.S2 – Fig.3 |
|  |  |  |  |  |  |  |
| C3_R35A03-R29G11 | C2+C3_R20C11-R48D11 | 0.3737 | 0.4148 | 0.2318 | 0.7642 | Fig.3 – Fig.3 |
| C3_R26H02-R29G11 | C2+C3_R20C11-R48D11 | 0.7861 | 0.3802 | 0.7262 | 0.0767 | Fig.S2 – Fig.3 |

**Video 1 pBDPGAL4U__shi_Ring.avi Example for control line (pBDPGAL4U>*shi^ts^*) at 34°C in the ring gap assay.** In the course of the experiment the flies leave the centre. The video plays at 30x recording speed.

**Video 2 R24D10__shi_Ring.avi Example for a “clumsy” line close to the “very clumsy” boundary.** Example for R24D10>*shi^ts^* at 34°C in the ring gap assay. All flies fall into the gaps within the first 90 seconds. The video plays at 30x recording speed.

**Video 3 R61H03__shi_Ring.avi Example for a “overcautious” line.** Example for R61H03>*shi^ts^* at 34°C in the ring gap assay. Most flies do not cross the 3.0 mm gap. The video plays at 30x recording speed.

**Video 4 R24D10__shi_HS_2mm.avi.** Example for R24D10>*shi^ts^* at 34°C at a single 2.0mm gap. The fly has problems in walking and already fails at a 2.0 mm gap. The video is slowed down 7x.

**Video 5 R23D06__shi_HS_3.5mm.avi.** Example for R23D06>*shi^ts^* at 34°C failing at a single 3.5 mm gap. The right part of the video shows a zoomed in view. The video is slowed down 7x.

**Video 6 R22F08__shi_HS_5mm.avi.** Example for R22F08>*shi^ts^* at 34°C showing a climbing attempt at an insurmountable 5.0 mm gap. The video is slowed down 7x.

**Video 7 R68A11__shi_HS_4mm.avi.** This video shows a R68A11>*shi^ts^* at 34°C fly crossing a 4.0 mm gap. The video is slowed down 7x.
